# A Multicenter Phase I/II Study of Obatoclax Mesylate Administered as a 3- or 24-Hour Infusion in Older Patients with Previously Untreated Acute Myeloid Leukemia

**DOI:** 10.1371/journal.pone.0108694

**Published:** 2014-10-06

**Authors:** Aaron D. Schimmer, Azra Raza, Thomas H. Carter, David Claxton, Harry Erba, Daniel J. DeAngelo, Martin S. Tallman, Carolyn Goard, Gautam Borthakur

**Affiliations:** 1 Princess Margaret Cancer Centre, Toronto, Ontario, Canada; 2 Columbia University Medical Center, New York, New York, United States of America; 3 The University of Iowa, Iowa City, Iowa, United States of America; 4 Penn State, Hershey, Pennsylvania, United States of America; 5 University of Alabama at Birmingham, Birmingham, Alabama, United States of America; 6 Dana-Farber Cancer Institute, Boston, Massachusetts, United States of America; 7 Leukemia Service, Memorial Sloan-Kettering Cancer Center, Weill Cornell Medical College, New York, New York, United States of America; 8 The University of Texas MD Anderson Cancer Center, Houston, Texas, United States of America; University of Maryland, United States of America

## Abstract

**Purpose:**

An open-label phase I/II study of single-agent obatoclax determined a maximum tolerated dose (MTD) and schedule, safety, and efficacy in older patients (≥70 yr) with untreated acute myeloid leukemia (AML).

**Experimental Design:**

Phase I evaluated the safety of obatoclax infused for 3 hours on 3 consecutive days (3 h×3 d) in 2-week cycles. Initial obatoclax dose was 30 mg/day (3 h×3 d; n = 3). Obatoclax was increased to 45 mg/day (3 h×3 d) if ≤1 patient had a dose-limiting toxicity (DLT) and decreased to 20 mg/day (3 h×3 d) if DLT occurred in ≥2 patients. In the phase II study, 12 patients were randomized to receive obatoclax at the dose identified during phase I (3 h×3 d) or 60 mg/day administered by continuous infusion over 24 hours for 3 days (24 h×3 d) to determine the morphologic complete response rate.

**Results:**

In phase I, two of three patients receiving obatoclax 30 mg/day (3 h×3 d) experienced grade 3 neurologic DLTs (confusion, ataxia, and somnolence). Obatoclax was decreased to 20 mg/day (3 h×3 d). In phase II, no clinically relevant safety differences were observed between the 20 mg/day (3 h×3 d; n = 7) and 60 mg/day (24 h×3 d; n = 5) arms. Neurologic and psychiatric adverse events were most common and were generally transient and reversible. Complete response was not achieved in any patient.

**Conclusions:**

Obatoclax 20 mg/day was the MTD (3 h×3 d) in older patients with AML. In the schedules tested, single-agent obatoclax was not associated with an objective response. Evaluation in additional subgroups or in combination with other chemotherapy modalities may be considered for future study.

**Trial Registration:**

ClinicalTrials.gov NCT00684918

## Introduction

Acute myeloid leukemia (AML) is a heterogeneous hematologic malignancy that results from the clonal expansion of primitive myeloid precursor cells [1]. AML is the most common form of acute leukemia in adults, and has a high mortality. In the United States, a total of 14,590 new AML diagnoses were projected to occur in 2013, with an estimated 10,370 deaths [2]. AML is predominantly a disease of older adults with a median age at diagnosis of 66 years [3]. Compared with younger patients (<55 years), AML in older patients (≥55 years) is more frequently associated with a poor prognosis, in part due to decreased response rates and increased toxicity of standard induction chemotherapy. Consequently, there is an unmet need for therapies that provide efficacy and favorable tolerability in older patients with AML.

Development of small-molecule inhibitors specific for anti-apoptotic proteins is a novel approach to the treatment of hematologic cancers. Anti-apoptotic B cell-chronic lymphocytic leukemia/lymphoma 2 (Bcl-2) family members (Bcl-2, Bcl-XL, Bcl-w, Bcl-b, A1/Bfl-1, and Mcl-1) are overexpressed in many cancers and inhibit apoptosis by sequestering pro-apoptotic members of the family (BH3-only proteins, and Bax and Bak) [4], [5]. Importantly, emerging evidence suggests that anti-apoptotic Mcl-1 is critical for sustained survival and expansion of human AML and plays a role in drug resistance in this disease [6]. Since interactions between anti- and pro-apoptotic family members are mediated by the BH3 domain protein interaction motif [4], [5], small molecules that bind to the BH3 binding groove may induce apoptosis by inhibiting sequestration of pro-apoptotic factors [7].

Obatoclax mesylate (obatoclax, also known as GX15-070) is a novel anticancer therapeutic for hematologic malignancies and solid tumors. The compound, which acts as a BH3 mimetic, was developed as a pan-inhibitor of anti-apoptotic members of the Bcl-2 family, including Mcl-1, to trigger cell death [8], [9]. Preclinical investigations demonstrated that obatoclax induces apoptosis and reduces proliferation in AML cell lines and primary AML cells, in part by inhibition of Mcl-1 sequestration of Bax [10].

In phase I trials of single-agent obatoclax, antitumor activity was observed in several hematologic malignancies, including AML, myelodysplastic syndrome, and Hodgkin's and non-Hodgkin's lymphoma [11]–[14]. Although there were a limited number of objective responses in these early clinical studies, hematologic improvement was observed in a larger proportion of treated patients. One striking clinical response occurred in a 70-year-old woman with previously untreated AML who achieved a complete response (CR) after receiving 20 mg/m^2^ obatoclax as a 24-hour infusion [14]. Her CR was maintained over 8 months and suggested that a subset of treatment-naive patients with AML might benefit from obatoclax therapy.

Continuous infusion of obatoclax 60 mg/day for 3 days (24 h×3 d) in 2-week cycles has previously been evaluated in phase II trials in patients with myelofibrosis or Hodgkin's lymphoma [13], [15]. An accelerated 3-hour infusion, 3-day (3 h×3 d) regimen has not yet been evaluated in a 2-week cycle in patients with hematologic malignancies, nor have these regimens been formally compared. The objectives of this multicenter phase I/II study were to expand on previous experiences with obatoclax and to evaluate the dose and schedule of single-agent obatoclax for safety and efficacy in older patients with previously untreated AML.

## Patients and Methods

### Study Design

**Figure 1:**
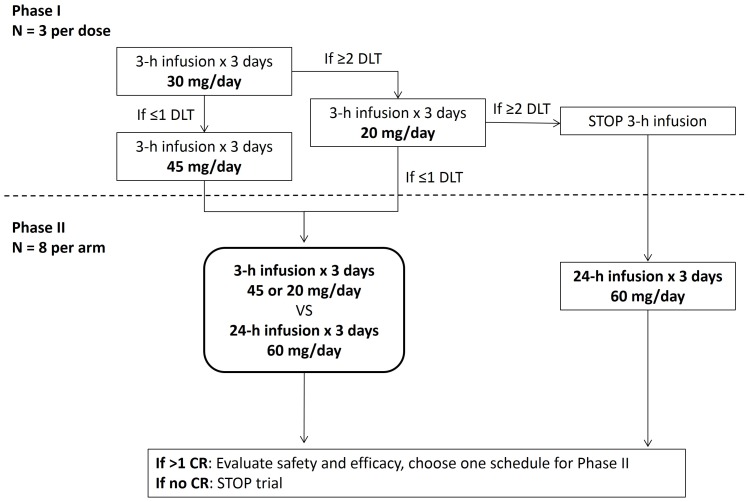
Study design. CR, complete response; DLT, dose-limiting toxicity.

An open-label, multicenter, phase I/II study was conducted. The protocol for this trial and supporting CONSORT checklist are available as supporting information; see **Checklist S1** and **Protocol S1**. The phase I portion of the study consisted of a nonrandomized safety evaluation, followed by a randomized phase II evaluation of different treatment schedules (**Figure 1**). The phase I safety evaluation assessed obatoclax as a 3-hour infusion over 3 consecutive days (3 h×3 d). Because the optimal schedule for obatoclax treatment was unknown, the phase II evaluation utilized a randomized open-label design to assess 3-hour or 24-hour infusion schedules, over 3 consecutive days (3 h×3 d or 24 h×3 d). The selected dose for the 24-hour infusion was the previously defined maximum tolerated dose (MTD) of 60 mg/day [16].

### Ethics Statement

The study was conducted in accordance with the October 2000 version of the Declaration of Helsinki, as well as Good Clinical Practice and International Conference on Harmonisation guidelines. An accredited institutional review board approved this study prior to its initiation and all patients provided informed written consent. This study was registered at clinicaltrials.gov (NCT00684918).

### Patient Eligibility

Patients at least 70 years of age with histologically confirmed AML were eligible to participate. In the phase I portion of the study, patients may have received one previous therapy. In the phase II portion of the study, no prior therapy for AML was allowed except for hydroxyurea. Additional eligibility requirements included Eastern Cooperative Oncology Group (ECOG) performance status ≤2 and normal hepatic and renal function (total bilirubin ≤2 mg/dL unless resulting from hemolysis; aspartate transaminase/alanine transaminase ≤2.5× institutional upper limit of normal; creatinine within normal institutional limits or creatinine clearance ≥50 mL/min/1.73 m^2^ for patients with creatinine levels above institutional normal).

Patients were excluded if they had a history of allergy to components of the formulated product. Comorbidities requiring patient exclusion included a history of seizure disorder or central nervous system leukemia or other symptomatic neurologic illness; uncontrolled systemic infection considered opportunistic, life-threatening, or clinically significant; symptomatic congestive heart failure; unstable angina pectoris; cardiac arrhythmia; significant pulmonary disease or hypoxia; psychiatric illness/social situation that would limit compliance with study requirements; or infection with human immunodeficiency virus.

### Treatments

Obatoclax mesylate (30 mg) was diluted with 5% dextrose, USP and a final concentration of 11.54% polyethylene glycol 300, 0.46% polysorbate 20 for intravenous (IV) infusion. Treatments were evaluated as depicted in **Figure 1**. In the phase I portion of the study, the first 3 patients enrolled received obatoclax 30 mg/day over 3 hours for 3 consecutive days (3 h×3 d). Dose-limiting toxicities (DLTs) were defined as grade ≥3 infusion-related neurologic adverse events (AEs) and nonhematologic AEs not responsive to symptom-directed therapy. If (during cycle 1) ≤1 of the 3 patients experienced a DLT, subsequent enrolled patients would receive 45 mg/day for 3 consecutive days (3 h×3 d). If, however, ≥2 of 3 patients experienced a DLT, an additional group of 3 patients would be enrolled and would receive 20 mg/day over 3 hours for 3 consecutive days (3 h×3 d). If ≤1 of 3 patients experienced a DLT at 20 mg/day, this dose would be utilized for the 3 h×3 d phase II study. If ≥2 of the 3 patients experienced a DLT at 20 mg/day, the 3 h×3 d schedule would be halted, and the phase II study would use only the 24 h×3 d schedule for obatoclax administration, which has previously been shown to be well tolerated and produced a CR in a patient with AML [14].

In the phase II portion of the study, patients were randomized (1∶1) into two arms. One arm received obatoclax at the dose identified from phase I at 3 h×3 d; the other arm received 60 mg/day (24 h×3 d). In both phases, obatoclax was administered in two 2-week cycles as induction therapy. Any patient achieving a CR could receive four additional treatment cycles as consolidation therapy every 2 weeks for a total of six cycles of obatoclax treatment. Patients who did not achieve CR after two cycles of obatoclax were to be removed from study.

Prophylaxis with H-1 and H-2 blockers was recommended prior to each cycle, given the known prevalence of acute hypersensitivity reactions associated with obatoclax exposure [14]. Full supportive care was offered to treat acute nausea, vomiting, or DLTs as appropriate, including anti-emetic prophylaxis, blood products, antibiotics, IV immunoglobulins, and hematopoietic growth factors.

### Endpoints and Assessments

#### Endpoints

Safety endpoints included number of DLTs, treatment-emergent AEs, including serious AEs, and clinical laboratory values. AEs were recorded according to the National Cancer Institute Common Terminology Criteria for Adverse Events, version 3.0 [17].

Clinical response was assessed using standard criteria [18]. The primary efficacy endpoint was rate of morphologic CR, or cytogenetic CR in patients with abnormal cytogenetics at baseline. Morphologic CR was defined as neutrophils >1000/µL, platelets >100,000/µL, and bone marrow blasts <5%. Cytogenetic CR was defined as neutrophils >1000/µL, platelets >100,000/µL, bone marrow blasts <5%, and normal cytogenetics. Additional efficacy endpoints included molecular CR, partial remission (PR), and morphologic leukemia-free state, and change in bone marrow blasts from baseline to post-induction therapy (day 28).

#### Schedule of assessments

AEs were recorded from baseline screening to 28 days after the last obatoclax dose. Complete blood counts (absolute neutrophil count, lymphocytes, monocytes, eosinophils, and basophils) were obtained on day 1 of each cycle, every 2–3 days for the first week of cycle 1, and on day 8 of each cycle. Physical and neurologic examinations, including vital signs, body weight, ECOG performance status, and serum chemistries were performed at baseline and on days 1 and 8 of each cycle. Chest radiographs, pulmonary function tests, and urinalysis were conducted at baseline and at the 28-day follow-up visit. An electrocardiogram was obtained at baseline, 30 minutes before the end of infusion on day 3 in cycle 1, and at the 28-day follow-up visit, and repeated as clinically indicated.

Bone marrow aspirates and biopsies were conducted at baseline, on day 28, after obatoclax consolidation therapy, and as clinically indicated. CR was documented by repeat bone marrow examination on day 28 or earlier. Bone marrow cytogenetics were assessed at baseline, repeated on occurrence of CR, and as clinically indicated.

### Statistical Analysis

Safety was assessed in all patients who received any amount of study drug per National Cancer Institute Common Terminology Criteria for Adverse Events, version 3. Efficacy was assessed in all patients with at least one post-baseline efficacy assessment. All outcomes were summarized descriptively. For categorical variables, summary tabulations of the number and percentage in each parameter were provided. For continuous variables, the mean, median, standard deviation, minimum, and maximum were presented. To determine the rate of morphologic CR, a two-stage design was used, powered to detect a CR rate of ≥15% against a non-interesting rate of 5%, with alpha  = 0.05 and a power of 90%. Stage 1 was to enroll 37 patients under this design, and if ≥3 patients achieved CR at the end of Stage 1, an additional 47 patients would be enrolled.

## Results

### Demographics and Patient Disposition

A total of 19 patients were enrolled in the study from March 2008 to March 2009; one patient did not receive obatoclax treatment. Demographics and disease characteristics were similar across all regimens (**Table 1**). Overall, the mean age was 81 years (range 72–90 years). The median time from AML diagnosis to study entry was 0.6 months (range 0–37 months). Most (56%) patients had a French-American-British classification of M2; the distribution of AML classification was similar for all regimens. Approximately 56% of patients had an abnormal karyotype; further details of the cytogenetic abnormalities were not available, thus precluding classification of cytogenetics into risk groups.

**Table 1:** Demographics and baseline characteristics of obatoclax-treated patients (N = 18).

	Phase I		Phase II		All (N = 18)
	30 mg/d (3 h×3 d) (n = 3)	20 mg/d (3 h×3 d) (n = 3)	20 mg/d (3 h×3 d) (n = 7)	60 mg/d (24 h×3 d) (n = 5)	
Median (range) age, years	83 (81–85)	74 (72–90)	82 (72–89)	80 (76–86)	81.5 (72–90)
Male, n (%)	1 (33)	1 (33)	3 (43)	3 (60)	8 (44)
ECOG PS, n (%)					
0	1 (33)	0	3 (43)	1 (20)	5 (28)
1	1 (33)	1 (33)	4 (57)	2 (40)	8 (44)
2	1 (33)	2 (67)	0	2 (40)	5 (28)
Median time since AML diagnosis, months (range)	1 (0.5–7.4)	1.2 (1–16.7)	0.45 (−0.2–0.6)	0.2 (0–37.2)	0.6 (−0.2–37.2)
AML classification, n (%)					
M1	1 (33)	0	3 (43)	0	4 (22)
M2	2 (67)	1 (33)	3 (43)	4 (80)	10 (56)
M3	0	1 (33)*	0	0	1 (6)
M4	0	1 (33)	0	1 (20)	2 (11)
Missing	0	0	1 (14)	0	1 (6)
Cytogenetics, n (%)					
Abnormal	1 (33)	3 (100)	3 (43)	3 (60)	10 (56)
Normal	1 (33)	0	2 (29)	2 (40)	5 (28)
Missing	1 (33)	0	2 (29)	0	3 (17)
Median leukocyte count (range), 10^3^/µL	1.3 (0.5–1.4)	29.2 (19.3–64.3)	4.3 (1.1–7.3)	2.8 (1.1–27.2)	4.25 (0.5–64.3)
Median platelet count (range), 10^3^/µL	23 (8–154)	126 (69–227)	63 (19–518)	63 (19–259)	66 (8–518)
Median hemoglobin (range), g/L	86 (86–102)	105 (99–110)	101 (86–117)	93 (90–100)	97.5 (86–117)
Median neutrophil count (range), 10^3^/µL	0.2 (0.2–0.2)^a^	1.95 (0.6–3.3)^b^	0.39 (0.1–2.6)^c^	6.98 (0.5–13.5)^d^	0.55 (0.1–13.5)^e^

*Classified as acute promyelocytic leukemia.

a Evaluated in 1 patient.

b Evaluated in 2 patients.

c Evaluated in 5 patients.

d Evaluated in 2 patients.

e Evaluated in 10 patients.

AML, acute myeloid leukemia; ECOG PS, Eastern Cooperative Oncology Group performance status.

**Figure 2:**
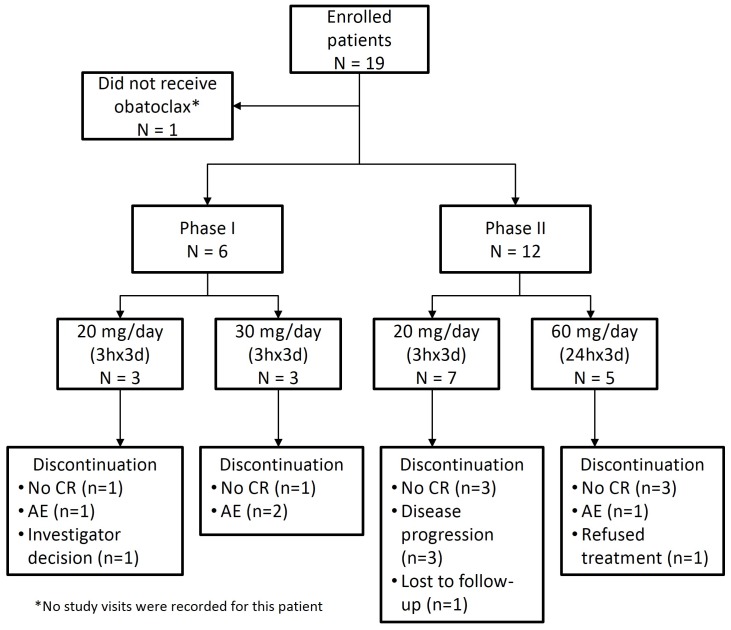
Patient disposition. AE, adverse event; CR, complete response.

Patient disposition is shown in **Figure 2**. The most common reasons for withdrawal from the study were failure to achieve CR following induction therapy (eight patients, 42%), adverse events (including DLT; four patients, 21%), and disease progression (three patients, 16%). Two patients were granted waivers for laboratory abnormalities (hyperuricemia) at baseline.

The number of treatment cycles administered in the phase I/II studies is shown in **Table 2** **.** Overall, the median number of cycles administered was two (range 1–11). In both the phase I and II studies, a total of 15 patients (83%) were treated for at least two cycles. Four patients also received additional treatment cycles as consolidation therapy as they experienced disease stabilization or decreased blast counts in the marrow, although these continued cycles were considered protocol deviations. Of these four patients, one patient received a total of eight cycles of therapy, two patients received a total of four cycles, and one patient received a total of 11 cycles.

**Table 2:** Study drug exposure in patients treated with obatoclax (N = 18).

	Phase I		Phase II		All (N = 18)
	30 mg/d (n = 3)	20 mg/d (n = 3)	3-h infusion (20 mg/d) (n = 7)	24-h infusion (60 mg/d) (n = 5)	
Median number of cycles (range)	2 (1–2)	4 (4–8)	2 (2–11)	2 (1–2)	2 (1–11)
Total cycles, n (%)					
1	3 (100)	3 (100)	7 (100)	5 (100)	18 (100)
2	2 (67)	3 (100)	7 (100)	3 (60)	15 (83)
3	0	3 (100)	1 (14)	0	4 (22)
4	0	3 (100)	1 (14)	0	4 (22)
5	0	1 (33)	1 (14)	0	2 (11)
6	0	1 (33)	1 (14)	0	2 (11)
7	0	1 (33)	1 (14)	0	2 (11)
8	0	1 (33)	1 (14)	0	2 (11)
>9	0	0	1 (14)	0	1 (6)

### Safety

In the phase I safety study, two of three patients treated with 30 mg/day (3 h×3 d) experienced grade 3 neurologic events that led to discontinuation of treatment and were classified as DLTs (**Table 3**). The first patient experienced grade 3 somnolence (day 1) and grade 3 confusion. This patient also experienced dizziness, mood alteration, and speech disorder during infusion (all grade <3; **Table 4**). The second patient experienced grade 3 ataxia and grade 1 confusion, euphoria, and somnolence on day 1 of cycle 1. In both patients, neurologic DLTs were assessed as definitely related to study drug and resolved within 24 hours. The third patient in this group also experienced euphoria and ataxia (grade <3) and discontinued due to leukemic infiltrate. One serious AE (grade 3 neutropenic fever) was reported for this regimen, but was judged to be unrelated to study drug (**Table 3**).

**Table 3:** Summary of dose-limiting toxicities and serious adverse events.

	Phase I		Phase II	
	30 mg/d (n = 3)	20 mg/d (n = 3)	3-h infusion (20 mg/d) (n = 7)	24-h infusion (60 mg/d) (n = 5)
Dose-limiting toxicity	Grade 3 somnolence/confusion (1); grade 3 ataxia (1)	0	0	0
Serious TEAE, n (grade, attribution)				
Febrile neutropenia	1 (gr 3, NR)		1 (gr 3, NR)	
Atrial fibrillation			2 (gr 3, NR; gr 3, NR)	
Acute myocardial infarction			1 (gr 3, NR)	1 (gr 4, PS)
Cough			2 (gr 1, NR; gr 2, PS)	
Catheter site infection			1 (gr 3, NR)	1 (gr 3, NR)
Cytokine release syndrome		1 (gr 2, PR)		
Pneumonia		1 (gr 3, NR)	1 (gr 3, NR)	
Acute sinusitis			1 (gr 1, NR)	
Dyspnea			1 (gr 2, NR)	
Fatigue				1 (gr 5, NR)
Dizziness				1 (gr 3, PR)

AE, adverse event; DLT, dose-limiting toxicity (DLTs were defined as grade ≥3 infusion-related neurologic AEs and nonhematologic AEs not responsive to symptom-directed therapy); PR, probably related; PS, possibly related; NR, not related; TEAE, treatment-emergent adverse event.

**Table 4:** Treatment-emergent adverse events occurring in more than one patient.

	Phase I 20 mg/d (n = 3)		Phase I 30 mg/d (n = 3)		Phase II 3-h infusion (20 mg/d) (n = 7)		Phase II 24-h infusion (60 mg/d) (n = 5)		All (N = 18)	
n	All grade	Grade ≥3	All grade	Grade ≥3	All grade	Grade ≥3	All grade	Grade ≥3	All grade	Grade ≥3
Euphoria	3	0	2	0	6	1	1	0	12	1
Somnolence	0	0	2	2	4	1	2	0	8	3
Ataxia	0	0	3	1	3	0	1	1	7	2
Dizziness	1	0	1	0	2	1	1	1	5	2
Confusion	0	0	2	1	1	0	2	0	5	1
Constipation	1		0		1		3		5	
Fever	0	0	0	0	3	1	2	0	5	1
Diarrhea	1		1		1		1		4	
Peripheral edema	0		0		2		2		4	
Febrile neutropenia	0	0	1	1	2	2	1	1	4	4
Disorientation	2		0		1		0		3	
Cough	0		1		2		0		3	
Unsteady gait	0		1		2		0		3	
Insomnia	1		0		2		0		3	
Dyspnea	0		0		1		2		3	
Hypoxia	0	0	0	0	3	1	0	0	3	1
Dysarthria	1		0		2		0		3	
Fatigue	0	0	0	0	2	0	1	1	3	1
Headache	0		0		2		1		3	
Ecchymosis	0		0		3		0		3	
Hypotension	0		0		1		1		2	
Tachycardia	0		0		2		0		2	
Cardiac murmur	0		0		2		0		2	
Atrial fibrillation	0	0	0	0	2	2	0	0	2	2
Pneumonia	1	1	0	0	1	1	0	0	2	2
Loose stool	1		0		1		0		2	
Gingival pain	0		1		1		0		2	
Cytokine release syndrome	1		0		1		0		2	
Abnormal breath sounds	0		0		1		1		2	
Acute MI	0	0	0	0	1	1	1	1	2	2
Dry mouth	0		0		1		1		2	
Crackles (lung)	0		0		1		1		2	
Thrush	0		0		2		0		2	
Hypocalcemia	0		1		1		0		2	
Hypokalemia	0		1		1		0		2	
Slurred speech	0		0		2		0		2	
Agitation	0	0	0	0	1	1	1	0	2	1
Muscular weakness	0		0		1		1		2	
Anxiety	0		1		1		0		2	

Per protocol, the obatoclax dose in the 3 h×3 d regimen was decreased to 20 mg/day in a subsequent cohort of three patients. No additional DLTs were observed in this group and only one grade 3 serious AE (pneumonia) was reported and was considered unrelated to study drug. In addition, one patient experienced grade 2 cytokine release syndrome, which was also considered a serious AE (**Table 3**). Therefore, the 20 mg/day (3 h×3 d) regimen was chosen for the randomized phase II portion of the study for comparison with obatoclax 60 mg/day (24 h×3 d), as previously defined in a small study of 18 patients [16].

In the phase II study, most patients receiving either obatoclax 20 mg/day (3 h×3 d) or 60 mg/day (24 h×3 d) experienced mild to moderate (grade <3), transient, neurologic AEs such as euphoria, somnolence, ataxia, dizziness, and confusion (**Table 4**). AEs of grade ≥3 that were reported in more than one patient included febrile neutropenia (n = 3), dizziness (n = 2), atrial fibrillation (n = 2), and acute myocardial infarction (n = 2). The events of dizziness, atrial fibrillation, and acute myocardial infarction were considered to be at least possibly related to obatoclax. Acute myocardial infarction resulted in treatment discontinuation in one patient.

Combining both the phase I and II components of the study, all 18 (100%) patients who received obatoclax experienced at least 1 AE, the most common of which were neurologic (n = 14; 77.8%) or psychiatric (n = 16; 88.9%); most were transient and mild, and resolved without sequelae. Ten patients experienced serious AEs; details are provided in **Table 3**. Evidence for trends between severity of AEs (grade ≥3) and dose or schedule was not observed (**Table 4**). AEs (any grade) with the highest reported incidence included euphoria (67%), somnolence (44%), and ataxia (39%). Somnolence was the most commonly reported grade ≥3 AE (17%). The most common grade ≥3 toxicities based on laboratory data were leukocytosis and thrombocytopenia (each n = 9). No clinically meaningful differences were observed across regimens for laboratory reports of grade ≥3 hematologic findings.

Two patients died during the study for reasons unrelated to obatoclax administration. One patient on the 60 mg/day (24 h×3 d) regimen died on day 23 of the study from progressive disease. The second patient, receiving 20 mg/day (3 h×3 d), died on day 41; the cause of death is unknown. An additional six patients died more than 30 days after the last obatoclax dose. Causes of death in these patients were progressive disease (n = 2), sepsis (n = 1), and unknown (n = 3).

### Efficacy

CR was not achieved with obatoclax induction. However, three patients on the 20 mg/day (3 h×3 d) regimen demonstrated a 7% to 17% decrease in bone marrow blast percentage between the baseline assessment and the end of cycle 2 (**Table 5**). It is noteworthy that two of these patients with decreased marrow blasts also demonstrated an increase of 33% to 57% in neutrophil count. Of these three patients, one patient in phase I receiving obatoclax 20 mg/day (3 h×3 d arm) had a decrease in marrow blasts from 27% to 10% with increased neutrophils from 9344×10^3^/µL to 14,700×10^3^/µL. This patient received eight cycles of study treatment and withdrew from the study on the advice of the investigator. One additional patient in the phase II portion of the study had increased neutrophil (pre-treatment: 230×10^3^/µL; end of cycle 2: 2500×10^3^/µL) and platelet counts (pre-treatment: 73×10^3^/µL; end of cycle 2: 250×10^3^/µL) without a significant change in the marrow blasts. This patient remained stable and received 11 cycles of obatoclax.

**Table 5:** Improvement in marrow blast count, neutrophil count, and platelet count in patients receiving obatoclax.

Patient	Treatment (phase)	AML Classification	Blast count, % (aspirate)		Neutrophils, ×10^3^/µL		Platelets, ×10^3^/µL	
			Base-line	End Cycle 2	Baseline	End Cycle 2	Baseline	End Cycle 2
04.002*	20 mg/d (I)	M4	27	10	9344	14,700	227	206
04.003	20 mg/d (I)	M3	27	20	3300	4400	126	139
05.002	20 mg/d (II)	M1	33	23	390	360	49	47

*Received eight cycles of obatoclax.

AML, acute myeloid leukemia.

## Discussion

The results of the current study demonstrate that the safety profile of obatoclax administered for 3 consecutive days (every 2 weeks) by 3-hour or 24-hour infusion was generally mild and similar to previous reports [11]–[14]. AEs were typically transient, neurologic, or psychiatric findings that resolved without sequelae. Based on the safety profile, 20 mg/day was determined to be the MTD of obatoclax when administered over 3 hours/day for 3 consecutive days in older AML patients. Two patients treated with the 30 mg/day (3 h×3 d) regimen experienced DLTs consisting of grade 3 confusion, somnolence, or ataxia, which led to premature discontinuation and selection of the 20 mg/day dose for further evaluation. In the phase II comparison of obatoclax 20 mg/day (3 h×3 d) to the previously evaluated 60 mg/day (24 h×3 d) regimen, both dosing schedules demonstrated similar safety profiles.

The mechanism(s) underlying the development of neurologic or psychiatric symptoms is uncertain. However, it is plausible that these symptoms represent an on-target effect, since Bcl-2 promotes neuron survival and Bcl-XL plays a role in synaptic plasticity [19], [20]. Alternatively, the neurologic and psychiatric effects of obatoclax may reflect binding to targets other than Bcl-2 family members.

Based on laboratory data, grade ≥3 leukocytosis and thrombocytopenia occurred in 50% of patients in this study. These hematologic abnormalities are likely related to underlying disease rather than obatoclax as they were present at baseline. However, it should be noted that inhibition of Bcl-XL by obatoclax may result in thrombocytopenia. Because patients also received platelet transfusions during the study as supportive care, the impact of obatoclax on platelet production may have been obscured.

Six patients experienced cardiac events during this study, four of which were assessed as at least possibly related to the study drug. The causal relationship with ischemic cardiac events, if any, is unclear because patients in this study were older with multiple comorbidities. In other clinical studies evaluating obatoclax, QTc prolongation has been reported by automated electrocardiogram. In a study of patients with relapsed small cell lung cancer, the interval between obatoclax doses was extended to 3 days in a single patient who experienced QTc prolongation during the first cycle [14], [21]. In another phase I dose escalation study in advanced hematologic malignancies, grade 3 QTc prolongation was observed in 3 patients, but was confounded by the presence of QTc prolongation at baseline [14]. Notably, an imbalance in the ratio of anti-apoptotic to pro-apoptotic Bcl-2 proteins appears causal in the development of cardiovascular disease, including ischemic heart disease [22], so relationship to study drug cannot be excluded.

In the current study, four patients had a clinical response of stable disease and were treated for up to 11 cycles. We did not observe a CR in this study. This contrasts with our previous report of a CR achieved with single-agent obatoclax (20 mg/m^2^ over 24 hours) in one older treatment-naive patient with AML with a mixed-lineage leukemia (MLL) t(9;11) translocation [14]. Although such dramatic single-agent activity in previously untreated AML was not confirmed by our data, it is possible that MLL-associated leukemia may be particularly sensitive to Bcl-2 family inhibitors. Preclinical studies have shown that inhibition of MLL expression using siRNA corresponded with reduced Bcl-XL levels and leukemic proliferation that may be mediated by HoxA9 [23], [24]. Thus, Bcl-2 proteins may play an important role in the proliferation of MLL-associated leukemia. In addition, a limitation of our study is that pharmacodynamic activity or pharmacokinetic parameters were not assessed. Integrating these evaluations in future clinical trials of obatoclax may provide further insight into the clinical potential of obatoclax as a single agent.

In addition to obatoclax, several other small-molecule BH3 mimetics are under investigation. ABT-737 and ABT-263 (navitoclax), for example, bind three of six Bcl-2 family members with high affinity [25], [26]. Navitoclax has been evaluated in phase I trials in lymphoid malignancies [27], [28]. However, these inhibitors do not bind Mcl-1 with as high affinity, and their therapeutic potential is constrained by dose-limiting thrombocytopenia associated with potent Bcl-XL inhibition in platelets [29]. Obatoclax was developed as a promiscuous Bcl-2 family inhibitor and also inhibits Mcl-1, which is essential for development and sustained growth of AML [6]. Follow-on analysis of the correlation between anti-apoptotic Bcl-2 proteins and obatoclax response was not conducted in this study, and will be important to include in future phase II evaluations.

It is conceivable, given their mechanism of action, that Bcl-2 family inhibitors might be most active in combination with other inducers of cell death. For example, obatoclax induced apoptosis in OCI-AML3 leukemic cells when used in combination with ABT-737 and synergistically induced apoptosis in combination with cytosine arabinoside in leukemic cell lines and in primary AML samples [10]. Preclinical investigations also suggest that obatoclax potentiates the effect of established drugs in AML [30]–[32], and several clinical trials of obatoclax combined with conventional chemotherapeutic agents have been completed or are ongoing in a range of solid tumors and hematologic malignancies (e.g., NCT00612612, NCT00521144 at clinicaltrials.gov).

In conclusion, based on the safety profile described in this study, 20 mg/day is the MTD of obatoclax when administered by 3-hour infusion over 3 consecutive days in an older AML population, and it has similar tolerability to a 60 mg/day (24 h×3 d) regimen. Although the current study does not support the efficacy of obatoclax as a single agent in an unselected group of treatment-naive AML patients, additional studies may reveal activity in select subgroups, particularly in combination with other chemotherapeutics.

## Supporting Information

Protocol S1 **Study protocol for open-label phase I/II study of single-agent obatoclax in older patients (≥70 yr) with untreated acute myeloid leukemia (AML).**
(PDF)

Checklist S1 **CONSORT checklist.**
(DOC)
