# Supplementary material for: A Multicenter Phase I/II Study of Obatoclax Mesylate Administered as a 3- or 24-Hour Infusion in Older Patients with Previously Untreated Acute Myeloid Leukemia
Source: PLoS One. 2014 Oct 6;9(10):e108694. doi: 10.1371/journal.pone.0108694 (PMC4186779; doi:10.1371/journal.pone.0108694)
Supplement: Protocol S1 — Study protocol for open-label phase I/II study of single-agent obatoclax in older patients (≥70 yr) with untreated acute myeloid leukemia (AML). (PDF) [file pone.0108694.s001.pdf]

OBATOCLAX MESYLATE
(GX15-070MS)

PROTOCOL GEM016

A MULTICENTER, OPEN-LABEL, 2-STAGE, PHASE II STUDY OF SINGLE-AGENT OBATOCLAX MESYLATE ADMINISTERED FOR 3 CONSECUTIVE DAYS EVERY 2 WEEKS TO OLDER PATIENTS WITH PREVIOUSLY UNTREATED ACUTE MYELOID LEUKEMIA (AML)

Final Issue/Report Date: 20 December 2007

Confidentiality Statement

The information in this document contains trade secrets and commercial information that are privileged or confidential and may not be disclosed unless such disclosure is required by applicable law or regulations. In any event, persons to whom the information is disclosed must be informed that the information is *privileged* or *confidential* and may not be further disclosed by them. These restrictions on disclosure will apply equally to *all* future information supplied to you, which is indicated as *privileged* or *confidential.*

PROTOCOL APPROVAL PAGE

I have read and approve this protocol. I assure that this study will be conducted according to all stipulations of the protocol, including all statements regarding confidentiality.

Project Physician: ______________________

Mark S. Berger, MD
Vice President of Clinical Development

Reviewer: ______________________

Mark J. Mariani
Senior Director
Regulatory Affairs and Compliance

Approval: /s/
____

CONTACT INFORMATION

Sponsor: Gemin X, Inc.
400 Chesterfield Parkway
Suite 100
Malvern, PA 19355
610-640-5735

IND No: 66,822

Table 1: Emergency Contact Information

**Role in Study**	**Name**	**Address and Telephone number**
Clinical Study Leader	Linda Dunton	Phone: 610-640-5735, ext 14<br>Mobile: 302-743-9092<br>Fax: 610-640-5737
Responsible Physician	Mark S. Berger, MD	Phone: 610-640-5735, ext 25<br>Mobile: 610-506-7309<br>Fax: 610-640-5737
Drug Safety Physician	Mark S. Berger, MD	Phone: 610-640-5735, ext 25<br>Mobile: 610-506-7309<br>Fax: 610-640-5737
24-Hour emergency contact	Gemin X	Pager: 888-595-9738

INVESTIGATOR AGREEMENT PAGE

I agree:

- To assume responsibility for the proper conduct of the study at this site.
- To conduct the study in compliance with this protocol, any future amendments, and with any other study conduct procedures provided by Gemin X, Inc. (hereafter referred to as Gemin X).
- Not to implement any changes to the protocol without agreement from the Sponsor and prior review and written approval from the Institutional Review Board (IRB), except where necessary to eliminate an immediate hazard to the patients, or for administrative aspects of the study (where permitted by all applicable regulatory requirements).
- That I am thoroughly familiar with the appropriate use of the investigational product, as described in the protocol, and any other information provided by the Sponsor including, but not limited to, the following: the current Investigator's Brochure (IB) or equivalent document and IB supplement (if applicable).
- That I am aware of, and will comply with, "Good Clinical Practices" (GCP) and all applicable regulatory requirements.
- To ensure that all persons assisting me with the study are adequately informed about the investigational product and of their study-related duties and functions as described in the protocol.
- That I have been informed that certain regulatory authorities require the Sponsor to obtain and supply, as necessary, details about the investigator's ownership interest in the Sponsor or the investigational product, and more generally about his/her financial ties with the Sponsor. Gemin X will use and disclose the information solely for the purpose of complying with regulatory requirements.

Hence I:

- Agree to supply Gemin X with any necessary information regarding ownership interest and financial ties (including those of my spouse and dependent children);
- Agree to promptly update this information if any relevant changes occur during the course of the study and for 1 year following completion of the study; and
- Agree that Gemin X may disclose any information it has about such ownership interests and financial ties to regulatory authorities.

____________________	____________________	__________
Investigator Signature	Printed Name	Date

1. SYNOPSIS

**Name of Sponsor/Company:**<br>Gemin X, Inc.	
**Name of Investigational Product:**<br>Obatoclax mesylate	
**Name of Active Ingredient:**<br>Obatoclax mesylate (GX15-070MS)	
**Title of Study:** A Multicenter, Open-Label, 2-Stage, Phase II Study of Single-Agent Obatoclax Mesylate Administered for 3 Consecutive Days Every 2 Weeks to Older Patients with Previously Untreated Acute Myeloid Leukemia (AML)	
**Study centers:**<br>5-9 study centers for the pilot safety and schedule evaluation and 10-80 study centers for the Phase II portion in North America and Europe	
**Studied period:**<br>Approximate duration of study: 3-12 months<br>Estimated date first patient enrolled: 15 March, 2008<br>Estimated date last patient completed: 15 March, 2009	**Phase of development:**<br>Phase II (preceded by pilot safety evaluation and pilot schedule evaluation)
**Study rationale:**<br>Considerable attention has been devoted to the needs of older patients with AML. The incidence of AML increases with age, and AML in older patients is more often preceded by myelodysplastic syndromes (MDS). Compared to AML in younger patients, AML in older patients is more frequently associated with unfavorable cytogenetics and with overexpression of multidrug resistance transporters. Furthermore, patient-related factors such as organ dysfunction and compromised performance status also contribute to lower complete remission (CR) rates and increased mortality within 30 days of induction chemotherapy in older patients. As a result, there is broad consensus that for many older patients with AML the risks of standard induction chemotherapy outweigh the benefits.<br><br>Single agent trials of obatoclax have been completed utilizing a variety of intravenous (IV) schedules. For short infusion durations (1-3h), dose limiting toxicities (DLTs) have been associated with somnolence, ataxia, and euphoria occurring during and shortly after the infusion. These CNS toxicities are of reduced frequency and intensity when 24-hour infusions are utilized. Clinical studies comparing the efficacy of the 3-hour and 24-hour obatoclax schedules have not been performed.<br><br>Obatoclax has displayed single agent antitumor activity in 3 clinical Phase I trials including patients with MDS, refractory chronic lymphocytic leukemia, and non-Hodgkin's lymphoma. In addition, dramatic evidence of benefit was observed in 1 patient with AML. A 70-year-old woman with a prior history of adjuvant anthracycline chemotherapy for breast cancer later developed AML with an 11q23 translocation. She was treated with obatoclax 20 mg/m^2 over 24 hours and achieved a cytogenetic CR with complete hematological recovery 8 days after the first infusion, with no significant toxicities. This CR was maintained for over 8 months during which obatoclax treatment was continued.	

The current study is designed to fully evaluate the therapeutic potential of obatoclax in older patients with previously untreated AML as an alternative to initial standard induction chemotherapy. The study is also designed to compare the efficacy and safety profiles of obatoclax administered for 3 consecutive days by 3-hour or 24-hour infusion in a pilot schedule evaluation, in order to determine which schedule of obatoclax to utilize in the Phase II portion of the study.

Objectives:

Primary

- Determine the rate of morphologic CR of obatoclax single-agent therapy in older patients with previously untreated AML.

Secondary

- Based on the safety and efficacy profile of obatoclax in the pilot schedule evaluation, determine whether 3-hour or 24-hour obatoclax infusions should be utilized in the Phase II portion of the trial.
- Describe the duration of CR following treatment with obatoclax.
- Describe the safety profile of obatoclax administered every 2 weeks to older patients with previously untreated AML.
- Describe the mortality rate within 30 days of the first dose of obatoclax.
- Describe the utilization of red blood cell and platelet transfusions following the start of obatoclax.
- Characterize the pharmacokinetic (PK) profile of obatoclax administered every 2 weeks to older patients with previously untreated AML.
- Characterize baseline expression of bcl-2 family members in leukemic cells to investigate the hypothesis that increased expression of specific pro-survival family members or decreased expression of bax and bak correlates with response to obatoclax.

Methodology:

This is a multicenter, open-label, 2-stage Phase II study of single-agent obatoclax mesylate administered for 3 consecutive days every 2 weeks in older patients with previously untreated AML. The 2-stage Phase II portion of the study is preceded by a pilot safety evaluation and a pilot schedule evaluation. Since the schedules for obatoclax administration evaluated previously do not include administering obatoclax by 3-hour infusion for 3 consecutive days, the pilot safety evaluation will first be conducted to evaluate the safety parameters of this schedule at a stepped-down dose in 3 patients. Then, the pilot schedule evaluation will be conducted to select the schedule to be evaluated (3-hour vs. 24-hour infusion; each administered for 3 consecutive days). The study design logic diagram is illustrated in Appendix 1, section 19.1.

During the first cycle of treatment (Cycle 1, Days 1-3), samples for PK analysis will be collected at appropriate time points throughout and beyond the infusional period. Therefore, overnight hospitalization will be required. Subsequent infusions may be administered on an outpatient basis. No investigational or commercial agents or therapies other than those described herein may be administered with the intent to treat the patient's malignancy.

For the nonrandomized pilot safety evaluation, the first 3 patients enrolled will receive a 3-hour infusion utilizing the stepped-down dose of 30 mg/day for 3 consecutive days (total dose of 90 mg over 3 days). During Cycle 1 of this pilot safety evaluation:

- If $\leq$1 of the 3 patients has a DLT, 45 mg/day for 3 consecutive days (total dose of 135 mg over 3 days) will be utilized for the 3-hour infusions in the subsequent pilot schedule evaluation.

- If ≥2 of the 3 patients have DLTs, an additional pilot safety evaluation dosage group will be enrolled to receive 20 mg/day as a 3-hour infusion for 3 consecutive days (total dose of 60 mg over 3 days).

Similar rules will apply to the 20 mg/day as a 3-hour infusion for 3 consecutive days dosage group if utilized in the pilot safety evaluation:

- If ≤1 of the 3 patients has a DLT, 20 mg/day as a 3-hour infusion for 3 consecutive days (total dose of 60 mg over 3 days) will be utilized for the 3-hour infusions in the pilot schedule evaluation.
- If ≥2 of the 3 patients have DLTs, the 3-hour infusion schedule will not be utilized further, and the pilot schedule evaluation will utilize only the 24-hour schedule for obatoclax administration.

In the pilot schedule evaluation portion of the trial, 16 patients will be randomized to treatment with 8 patients assigned to the group receiving 45 mg/day obatoclax administered as a 3-hour infusion for 3 consecutive days (total dose 135 mg over 3 days; dependent on the results of the pilot safety evaluation) and 8 patients assigned to the group receiving 60 mg/day obatoclax administered as a continuous infusion for 3 consecutive days (total dose 180 mg over 3 days), each to be repeated 2 weeks later as an initial induction therapy.

The first 3 patients enrolled into the pilot schedule evaluation portion of the trial receiving 45 mg/day over 3 hours will be evaluated for safety after Cycle 1. If ≤1 of these 3 patients has a DLT, then 45 mg/day will continue to be utilized for this arm of the pilot schedule evaluation with dose reductions for toxicities as needed. If ≥2 patients have DLTs during Cycle 1, then 30 mg/day will be utilized for the remainder of this arm of the pilot schedule evaluation portion of the trial. In this case, 8 additional patients would be enrolled into this arm.

If no CRs are observed in either the 3-hour or the 24-hour continuous infusion group of the pilot schedule evaluation, the trial will stop. If a CR is observed in at least 1 of the 8 patients in either group, the safety and efficacy profiles of the 3-hour and 24–hour infusion groups will be compared to determine which schedule of obatoclax administration to utilize in the Phase II portion of the trial.

The Phase II portion of the trial with a formal 2-stage design will enroll 37 patients into Stage 1 using the more favorable obatoclax administration schedule from the pilot schedule evaluation. If ≥3 of the 37 patients enrolled achieve a CR by the end of Stage 1, the trial will proceed to Stage 2, which will enroll an additional 47 patients for a total of up to 84 patients.

Patients achieving a CR (documented by a repeat bone marrow examination on Day 28) in any portion of this trial will receive 4 additional cycles of obatoclax as consolidation. Thus, patients achieving CR will receive a total of 6 cycles of obatoclax treatment. Patients who fail to achieve a CR following induction therapy (ie, Cycles 1 and 2) will be removed from study.

Number of patients (planned):
14-103

**Rationale for planned number of patients:**<br>Initially 3 patients will be enrolled into the pilot safety evaluation stepped-down 3-hour infusion dosage group utilizing 30 mg/day for 3 consecutive days (total dose 90 mg over 3 days). Then, 16 patients will be randomized into 1 of 2 cohorts (3-hour or 24-hour obatoclax infusion) and, if no CR is observed, the trial will stop. If ≥1 CR is observed in 1 of the 2 treatment regimens, the trial will proceed to the formal 2-stage design Phase II portion of the trial, which will enroll an additional 37 patients in Stage 1. At the end of Stage 1, if ≥3 CRs are noted among the 37 patients enrolled, the trial will proceed to Stage 2 which will enroll an additional 47 patients for a total of up to 84 in both Stages of the Phase II portion of the study.
**Diagnosis and main criteria for inclusion:**<br>Inclusion Criteria: 1) Histologically or cytologically confirmed AML; 2) No prior chemotherapy for AML with the exception that the patients enrolled in the pilot safety evaluation may have had 1 prior therapy; 3) Age ≥70 years; 4) Eastern Cooperative Oncology Group (ECOG) Performance Status ≤2; 5) Normal organ function defined as total bilirubin ≤2 mg/dL unless resulting from hemolysis, aspartate transaminase (AST[SGOT])/alanine transaminase (ALT[SGPT]) ≤2.5 x institutional upper limit of normal, creatinine within normal institutional limits OR creatinine clearance ≥50 mL/min/1.73 m^2 for patients with creatinine levels above institutional normal; 6) Because of the potential for unknown risks to an unborn child that could result from exposure to obatoclax, men with partners of child-bearing potential must agree to abstain from sexual activity that would result in pregnancy for the duration of study participation; 7) Willingness to submit to blood sampling for the planned PK analyses; 8) Ability to understand and willingness to sign a written informed consent form.<br><br>Exclusion Criteria: 1) Patients who have received or are receiving any other investigational or commercial agents or therapies administered with the intent to treat their malignancy, or patients who have had prior exposure to obatoclax (with the exception that the patients enrolled in the pilot safety evaluation may have had 1 prior therapy); 2) patients with history of allergic reactions attributed to components of the formulated product (PEG300 and polysorbate 20); 3) patients with history of seizure disorders or central nervous system leukemia; 4) patients with uncontrolled, intercurrent illness including, but not limited to, symptomatic neurological illness; active, uncontrolled, systemic infection considered opportunistic, life-threatening, or clinically significant at the time of treatment; symptomatic congestive heart failure; unstable angina pectoris; cardiac arrhythmia; significant pulmonary disease or hypoxia; or psychiatric illness/social situations that would limit compliance with study requirements; 5) Human immunodeficiency virus (HIV)-positive patients receiving combination anti-retroviral therapy.

Investigational product dosage and mode of administration:

Obatoclax (30 mg) is supplied as a lyophilized cake to be reconstituted with 5% Dextrose for Injection, USP (2 mL) and 8 mL of a diluent composed of 96.2% PEG 300 and 3.8% polysorbate 20. The desired dose of reconstituted obatoclax is to be further diluted with 5% Dextrose for Injection, USP and additional diluent to a total volume of 250 mL. The use of a soft non-di-2-(ethylhexyl) phthalate (DEHP) fluid path, an infusion set with polyethylene-lined tubing, and a non-DEHP pump segment fitted with a 0.22 µm filter are required. Central or peripheral venous catheters with polyurethane components are not recommended to be used with obatoclax as the formulated drug product may not be compatible with the catheter. Certain catheters composed of silicone components can be used (See Investigator's Brochure for details).

In the Phase II portion of the study, the maximum dose of obatoclax will be either a 45 mg flat dose (depending on results from the pilot safety evaluation) administered over 3 hours for 3 consecutive days (for a total of 135 mg over 3 days) or a flat dose of 60 mg administered over 24 hours for 3 consecutive days (for a total of 180 mg over 3 days). When obatoclax is administered by continuous infusion, the infusion bag of obatoclax will be changed for each 24-hour period as applicable.

Any patient who experiences DLTs may continue on therapy once the DLTs have resolved to <Grade 3 if the patient's dose is reduced (to 30 mg/day for the 3-hour 45 mg/day infusion regimen, to 20 mg/day for the 3-hour 30 mg/day infusion regimen, or to 45 mg/day for the 24-hour 60 mg/day infusion regimen).

Duration of treatment:

Patients achieving a CR (documented by a repeat bone marrow examination on Day 28 or earlier) in any portion of this trial will then receive 4 additional 3-day cycles of consolidation every 2 weeks (Cycles 3-6). Thus, patients achieving CR will receive a total of 6 cycles of obatoclax treatment.

Patients who fail to achieve a CR following the induction therapy (Cycles 1 and 2) will be removed from study.

Patients will be followed until death, and any subsequent anti-leukemic therapy will be documented.

Concomitant medications:

A cytokine-release syndrome has been reported during infusion of obatoclax. Prophylaxis with H-1 and H-2 blockers is recommended prior to each cycle of obatoclax. Full supportive care measures will be offered to treat any emerging DLTs.

Supportive care measures, including those directed at controlling symptoms resulting from hematological malignancies (blood products, antibiotics, IV immunoglobulins, hematopoietic growth factors, etc.), are allowed. However, any other treatment for the underlying malignant disease, with the exception of hydroxyurea, is prohibited, including other experimental therapies.

Criteria for evaluation:

Efficacy:

Response will be evaluated according to standard criteria. The following assessments will be conducted:

- Peripheral blood counts (at baseline and monitored throughout the study)
- Bone marrow aspirates and biopsies (at baseline and on Day 28, unless a CR has been confirmed earlier, after obatoclax consolidation therapy, and repeated as clinically indicated)
- Bone marrow cytogenetics (at baseline, repeated in case of CR and as clinically indicated)

All bone marrow aspirate and biopsy samples collected at baseline and during the study will be reviewed by an independent central pathologist.

Safety:

- Emerging adverse events ([AEs] monitored throughout the study)
- Physical and neurological examinations (at baseline and on Days 1 and 8 of each cycle)
- Vital signs (at baseline, on Days 1 and 8 of each cycle)
- Body weight (at baseline and on Days 1 and 8 of each cycle)
- Performance status (at baseline and on Days 1 and 8 of each cycle)
- Complete blood count (at baseline, on Day 1 of each cycle, every 2 to 3 days for the first week of the Cycle 1, and Day 8 of each cycle)
- Serum chemistry (at baseline and on Days 1 and 8 of each cycle)
- Urinalysis (at baseline and at the 28 day follow-up visit)
- Electrocardiogram ([ECG] at baseline, 30 minutes before the end of infusion on Day 3 in Cycle 1, and at the 28 day follow-up visit, to be repeated as clinically indicated)
- Chest radiographs (at baseline and at the 28 day follow-up visit)
- Pulmonary function tests (at baseline and 28 day follow-up visit)

Pharmacokinetics:

For all patients receiving 3-hour obatoclax infusions, 21 blood samples will be drawn at the following points, during Cycle 1 only:

- Day 1 timed from the start of the infusion: predose, 0.5, 1, 2.5, 3 (prior to end of infusion),
- Day 1 timed from the end of the infusion: 0.5, 1, 3, and 9 hours,
- Day 2: predose and 2.5 hours (30 minutes prior to end of infusion),
- Day 3 timed from the start of the infusion: predose, 0.5, 1, 2.5, 3 (prior to end of infusion),
- Day 3 timed from the end of the infusion: 0.5, 1, 3, 9, and 21 hours

For all patients receiving 24-hour obatoclax infusions, 14 blood samples will be collected during Cycle 1 only. Blood samples will be drawn at: pre-dose, 1, 4, 8, 22, 32, 46, 56, and 70 hours (timed from the start of the infusion). Following termination of the 72-hour infusion period of Cycle 1, additional samples will be drawn at 0.5, 1, 3, 9, and 21 hours, timed from the end of the infusion.

At each time point, 3 mL blood will be drawn into pre-chilled EDTA/K2 plastic tubes. For the 3-hour infusions, the total volume of blood sampled will be up to 63 mL over 3 days of dosing during Cycle 1. For the 24-hour infusions, the total volume of blood sampled will be up to 42 mL over 3 days of dosing during Cycle 1.

**Statistical methods:**<br>The Phase II portion of the trial will follow a 2-stage design powered to detect a true CR rate of ≥15% against a non-interesting rate of 5%, with an α=0.05 and a power of 90%. The formal 2-stage design will enroll 37 patients into Stage 1 using the more favorable obatoclax administration schedule from the pilot schedule evaluation. If at the end of Stage 1 ≥3 CRs occur among the 37 patients enrolled, the trial will proceed to Stage 2, which will enroll an additional 47 patients for a total of up to 84. A total of 8 or more CRs out of the 84 patients enrolled into Stages 1 and 2 would be required to conclude that the CR rate is significantly greater than 5% with over 90% confidence and that the confidence intervals for the true CR rate include 15%.<br><br>The demographic, exposure, safety (AEs and clinical laboratory evaluations), efficacy, and PK data will be summarized across subsets. Exploratory analysis of the relationships between PK parameters and population characteristics will be performed as appropriate.<br><br>The primary analysis of efficacy will be conducted on the rate of CR as determined by an independent central pathologist. Additional secondary efficacy analyses will be performed on the duration of CR, the rate of partial remission, and the utilization of red blood cell and platelet transfusions following the start of obatoclax. The rate of CR will be tabulated and accompanied by a 2-sided, 95% confidence interval. The duration of CR, defined as the duration in days from the date of first assessment of confirmed CR to the date of relapse or death, will be analyzed using Kaplan-Meier methods, with statistical assessment based on the log-rank statistic at the α=0.05 significance level. The rates of CR will be calculated for each of the stages of the Phase II portion of the study.

2. TABLE OF CONTENTS, LIST OF TABLES, AND LIST OF FIGURES

TABLE OF CONTENTS

1. SYNOPSIS 5
2. TABLE OF CONTENTS, LIST OF TABLES, AND LIST OF FIGURES 12
3. LIST OF ABBREVIATIONS AND DEFINITIONS OF TERMS 18
4. INTRODUCTION 20
4.1.1. Summary of Potential Risks to Human Subjects and Guidance to the Investigator 24
4.1.1.1. General Handling of Obatoclax 24
4.1.1.2. Nervous System Adverse Events 25
4.1.1.3. Respiratory System 25
4.1.1.4. Cytokine Release Syndrome 25
5. TRIAL OBJECTIVES AND PURPOSE 26
5.1. Primary objective 26
5.2. Secondary objectives 26
6. INVESTIGATIONAL PLAN 27
6.1. Overall Study Design and Plan: Description 27
6.1.1. Pilot Safety Evaluation 27
6.1.2. Pilot Schedule Evaluation 28
6.1.3. Phase II Portion of the Study 28
6.2. Study Assessments 31
6.2.1. Demographic and Baseline Assessments 31
6.2.2. Efficacy Assessments 31
6.2.3. Safety Assessments 31
6.2.3.1. Pregnancy 32
6.2.4. Translational Medicine Studies 32
7. SELECTION AND WITHDRAWAL OF SUBJECTS 33
7.1. Subject Inclusion Criteria 33
7.2. Subject Exclusion Criteria 33
7.3. Subject Withdrawal Criteria 34
8. TREATMENT OF SUBJECTS 35

8.1. Description of Study Drug 35
8.1.1. Definition of Dose-Limiting Toxicity 35
8.1.2. Obatoclax Administration 35
8.1.2.1. Pilot Safety Evaluation 35
8.1.2.2. Pilot Schedule Evaluation 35
8.1.2.3. Phase 2 Portion of the Study 36
8.1.3. Dosing Delays/Dose Modifications 36
8.1.4. Supportive Care Guidelines 36
8.1.5. Accidental Overdose 37
8.1.6. Duration of Therapy 37
8.2. Concomitant Medications 37
8.3. Treatment Compliance 37
8.4. Randomization and Blinding 37
8.5. Follow Up After Completion of Obatoclax Therapy or Withdrawal from Study 37
9. STUDY DRUG MATERIALS AND MANAGEMENT 38
9.1. Study Drug 38
9.2. Study Drug Packaging and Labeling 38
9.3. Study Drug Storage 38
9.4. Study Drug Preparation 38
9.4.1. Incompatibilities 38
9.4.2. Reconstitution 38
9.4.3. Dilution 39
9.5. Infusion Administration 40
9.6. Study Drug Accountability 41
9.7. Study Drug Handling and Disposal 41
10. ASSESSMENT OF EFFICACY 42
10.1. Response Criteria 42
10.2. Recurrence Criteria 42
10.3. Duration of Response 42
11. ASSESSMENT OF SAFETY 44
11.1. Safety Parameters 44
11.1.1. Definitions 44

11.2. Relationship to Study Drug 46
11.3. Intensity of Adverse Events 47
11.4. Recording Adverse Events 47
11.5. Reporting Adverse Events 47
11.5.1. Prompt Reporting of SAEs 47
11.5.2. Completion and Transmission of SAE Reports 48
11.5.3. Regulatory Reporting Requirements for SAEs 48
11.6. Follow Up of Adverse Events 49
11.6.1. Post-Study AEs and SAEs 50
11.6.2. SAEs Related to Study Participation 50
12. PHARMACOKINETIC ASSESSMENT 51
12.1. Blood Samples 51
12.2. Sampling Times 51
12.3. Technical Procedure 51
12.4. Bioanalytical Method 52
13. STATISTICS 53
14. DIRECT ACCESS TO SOURCE DATA/DOCUMENTS 55
14.1. Study Monitoring 55
14.2. Audits and Inspections 56
14.3. Institutional Review Board 56
14.4. Investigator Reporting Requirements 57
15. QUALITY CONTROL AND QUALITY ASSURANCE 58
16. ETHICS 59
16.1. Ethics Review 59
16.2. Ethical Conduct of the Study 59
16.3. Written Informed Consent 59
17. DATA HANDLING AND RECORDKEEPING 61
17.1. Inspection of Records 61
17.2. Retention of Records 61
17.3. Data Management 61
17.4. Study and Site Closure 62
17.5. Provision of Study Results and Information to Investigators 63
18. REFERENCES 64

19. APPENDICES 65
19.1. Appendix 1: Study Design Logic Diagram 65
19.2. Appendix 2: ECOG Performance Scale 66

LIST OF TABLES

Table 1: Emergency Contact Information....3
Table 2: Abbreviations and specialist terms....18
Table 3: IC50 Values for Obatoclax in Myeloid Leukemia Cell Lines....22
Table 4: Schedule of Study Assessments....29
Table 5: Dose Reconstitution....40
Table 6: Response Criteria in Acute Myeloid Leukemia....42
Table 7: Timeframes for Reporting All Serious Adverse Events....48

List of Figures:

Figure 1: Obatoclax Inhibits Growth in Myeloid Leukemia Cell Lines: Viability in AML Cell Lines at 72 Hours........22

Figure 2: Obatoclax Induces Apoptosis in OCI-AML3 Cells........22

Figure 3: Obatoclax Effect on CFU-Blast formation of primary AML samples........23

Figure 4: Obatoclax Induces Apoptosis in Primary AML Samples........23

Figure 5: Obatoclax Effect on CFU-Blast Formation in Normal Bone Marrow........23

3. LIST OF ABBREVIATIONS AND DEFINITIONS OF TERMS

Table 2: Abbreviations and specialist terms

Abbreviation or Specialist Term	Explanation
μL	microliter
μm	micrometer
μM	micromolar
AE	adverse event
ALT (SGPT)	alanine transaminase
AML	acute myeloid leukemia
AST (SGOT)	aspartate transaminase
AUC	area under the curve
BH	bcl-2 homology
°C	degrees Celsius
CBC	complete blood count
C_{max}	maximum concentration
CNS	central nervous system
Cl	clearance
CR	complete remission
CRF	case report form
CTCAE	common terminology criteria for adverse events
D5W	5% dextrose in sterile water
DEHP	di-2-(ethylhexyl) phthalate
dL	deciliter
DLT	dose-limiting toxicity
ECOG	Eastern Cooperative Oncology Group
ECG	electrocardiogram
EMD	extramedullary disease
°F	degrees Fahrenheit
FDA	United States Food and Drug Administration
GCP	Good Clinical Practices
HIPAA	Health Insurance Portability and Accountability Act

Table 2: Abbreviations and Specialist Terms (Continued)

Abbreviation or Specialist Term	Explanation
HIV	human immunodeficiency virus
IB	Investigator's Brochure
IC50	concentration of a substance that causes 50% inhibition
ICH	International Conference on Harmonization
IND	Investigational New Drug [Application]
IRB	Institutional Review Board
IV	intravenous
m^2	meters squared
MDS	myelodysplastic syndromes
MedDRA	Medical Dictionary of Regulatory Activities
mg	milligram
min	minute(s)
mL	milliliter
mRNA	messenger RNA
MS	mesylate salt
MSDS	material safety data sheet
MTD	maximum tolerated dose
NCI	National Cancer Institute
ng	nanogram
PEG	polyethylene glycol
OS	overall survival
pH	hydrogen ion concentration
PK	pharmacokinetic
SAE	serious adverse event
USP	United States Pharmacopeia
V	volume
V_{ss}	volume of distribution at steady state

4. INTRODUCTION

Considerable attention has been devoted to the needs of older patients with acute myeloid leukemia (AML). It is recognized that the incidence of AML increases with age and that AML in older patients is more often preceded by myelodysplastic syndrome, and is more frequently associated with unfavorable cytogenetics and with overexpression of multidrug resistance transporters [Appelbaum 2006, Tallman 2005]. Furthermore, patient-related factors such as organ dysfunction and compromised performance status also contribute to lowered complete remission (CR) rates and increased mortality within 30 days of induction chemotherapy [Appelbaum 2006]. As a result, there is broad consensus that a subset of elderly patients with AML exists for whom the risks of standard induction chemotherapy outweigh the benefits [Stone 2006, Tallman 2005].

Patients with newly diagnosed AML, if not treated, will progress rapidly to a fatal outcome. The treatments for AML are designed to be sufficiently aggressive to achieve complete remission because partial remission offers no substantial survival benefit. The goal of remission induction therapy in AML is to reduce the leukemia burden to a level undetectable by standard morphologic techniques. For almost 2 decades, the standard remission induction for AML has been a combination of 7 days of cytarabine at 100-200 mg/m^2 daily with 3 days of daunorubicin at 60 mg/m^2 daily (7 & 3 regimen) [Tallman 2005]. In patients who achieve complete remission, consolidation therapy is given. Salvage therapies are reserved for the time of relapse. With this 7 & 3 combination therapy, complete remission can be expected in approximately 50% to 75% of adult patients although only 20-30% achieve long-term disease-free survival [Tallman 2005].

Elderly patients with AML present a unique clinical entity. The efficacy of therapy in older patients with newly diagnosed AML is limited by a higher incidence of intrinsic resistance to chemotherapy and a reduced ability to tolerate both antileukemic therapy itself and the associated supportive care (e.g., nephrotoxic anti-infective therapy) [Appelbaum 2006, Taylor 1995]. Because of poor results with standard 7 & 3 therapy in older patients, clinical trials have evaluated the effects of lowering the dose of daunorubicin from 60 mg/m^2 to either 45 or 30 mg/m^2 for patients ≥60 years of age. Although this regimen was characterized by a 45% CR rate in patients >55 years of age, the median overall survival (OS) was only 8 months [Godwin 1998]. Attempts to improve on these results by utilizing other agents, such as mitoxantrone and etoposide, have been disappointing [Anderson 2002]. Patients ≥70 years of age or those ≥60 years of age with high-risk cytogenetics have a particularly poor prognosis, with median OS of 7.2 months and 3-year OS rates of 6% [Frohling 2006]. Thus although CR rates in older adults are approximately 50% in some studies, the overall survival rates are very poor, being <10% [Tallman 2005].

A high rate of early treatment-related mortality is a major contributor to the lower survival rates observed in elderly patients with poor-risk AML. Reduced tolerability and increased risk factors to induction chemotherapy, both of which are related to increasing age, represent a multifactorial risk/benefit outcome affected by duration and severity of treatment-induced myelosuppression, gastrointestinal mucositis, baseline organ dysfunction or co-existing medical conditions leading to organ malfunction, poor performance status, and pre-malignant conditions [Appelbaum 2006].

Retrospective analysis of 2657 AML patients ≥65 years of age by Menzin et al. indicated that only 30% of them received chemotherapy, with an inverse relationship between age and likelihood of receiving induction or palliative chemotherapy.

Anti-apoptotic members of the Bcl-2 family of cell-death regulators are up-regulated in many tumors of hematological and epithelial origin and provide the cancer cell with an inherent cell-survival mechanism that resists induction of cell death. [Cory 2002; Beauparlant 2003]. As a result, these cancers are refractory to apoptosis that is otherwise induced by the cancer-initiating signal itself (i.e., transforming oncogenes) or by many anti-cancer therapies. Anti-apoptotic Bcl-2 family members (BCL-2, BCL-XL, MCL-1, BCL-w, A1, and BCL-b) interact with pro-apoptotic members of the family (BH3-only proteins and Bax and Bak) and, when in excess, ultimately inhibit the ability of the pro-apoptotic members to induce the cell-death pathway. This, in turn, prevents the activation of a class of cysteine proteases called caspases, which cause apoptosis. Interactions between Bcl-2 family proteins occur through the docking of a BH3 domain on a pro-apoptotic member with a deep binding groove on the surface of anti-apoptotic members. Small-molecule drugs, such as obatoclax, that mimic the BH3 domain have the ability to bind to anti-apoptotic Bcl-2 family members and inhibit them, resulting in cancer-cell death. In contrast to cancer cells, where the inherent cancer-stress signals that specify cell death are inhibited by anti-apoptotic Bcl-2 family members, most cells of the body lack these inherent death signals. Therefore, inhibitors of Bcl-2 family proteins are predicted to be significantly less toxic to normal cells.

Obatoclax mesylate (GX15-070MS) is a small-molecule antagonist of the BH3-binding groove of the Bcl-2 family of proteins. *In vitro* studies of obatoclax have demonstrated inhibition of the growth of both cell lines and patient samples, and initial clinical studies of obatoclax have suggested that obatoclax may be active as a single-agent in AML.

In vitro studies have demonstrated that obatoclax as a single-agent inhibits the cell growth of AML cell lines (Figure 1 and Table 3) and induces apoptosis in AML cell lines (Figure 2) [Watt 2006]. Obatoclax inhibits CFU-blast colony formation in primary AML cells (Figure 3), and induces apoptosis in primary AML cells (Figure 4) [Watt 2006]. Obatoclax had limited effects on CFU-blast colony formation in 3 out of 4 normal bone marrows tested (Figure 5). Obatoclax also demonstrated synergy with cytarabine *in vitro* in AML cell lines [Watt 2006].

Figure 1: Obatoclax Inhibits Growth in Myeloid Leukemia Cell Lines: Viability in AML Cell Lines at 72 Hours

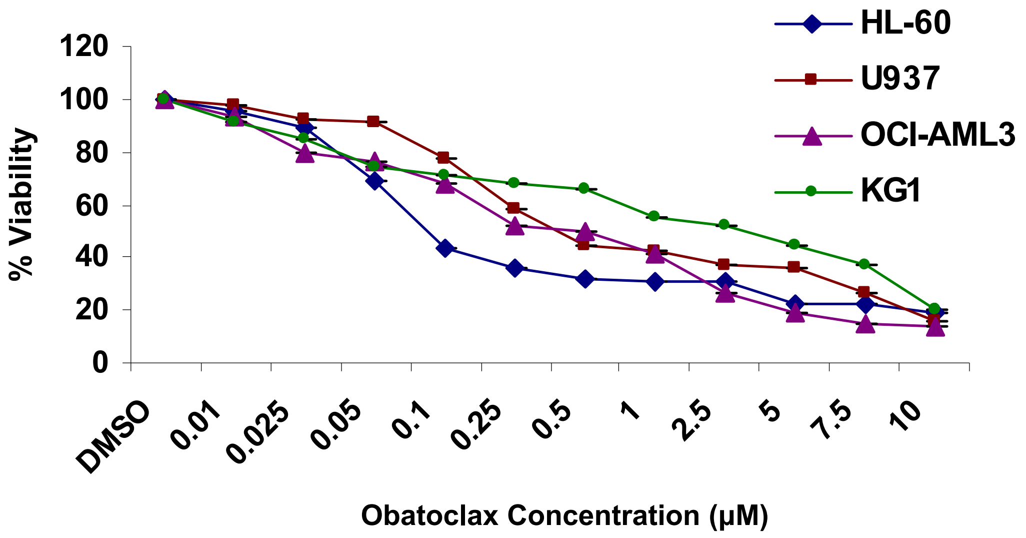

Table 3: IC50 Values for Obatoclax in Myeloid Leukemia Cell Lines

Cell Line	IC50 (µM)
HL-60	0.1
U937	0.5
OCI-AML3	0.5
KG1	2.5

IC50 = Concentration of a substance that causes 50% inhibition

Figure 2: Obatoclax Induces Apoptosis in OCI-AML3 Cells

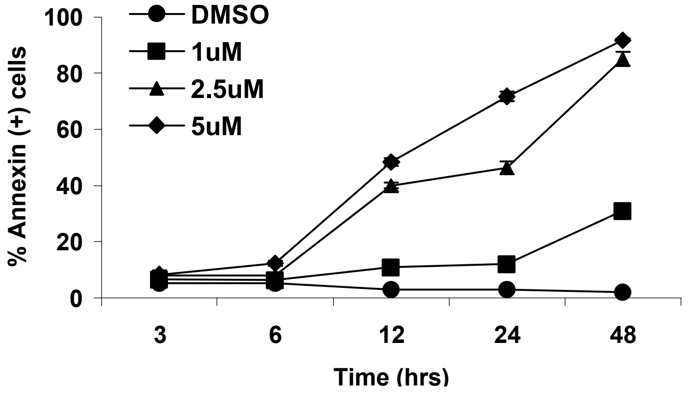

Figure 3: Obatoclax Effect on CFU-Blast formation of primary AML samples

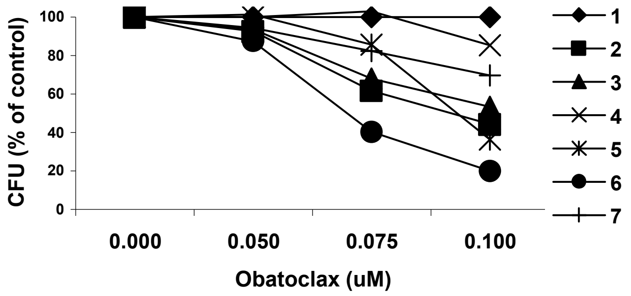

Figure 4: Obatoclax Induces Apoptosis in Primary AML Samples

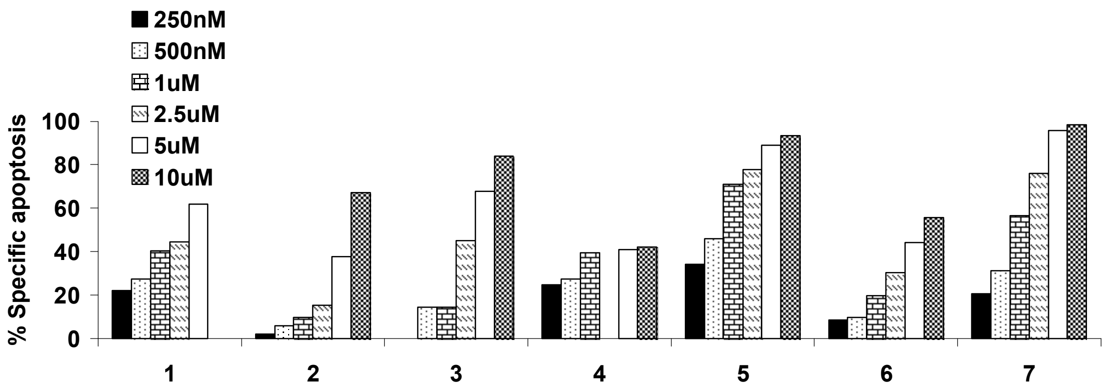

Figure 5: Obatoclax Effect on CFU-Blast Formation in Normal Bone Marrow

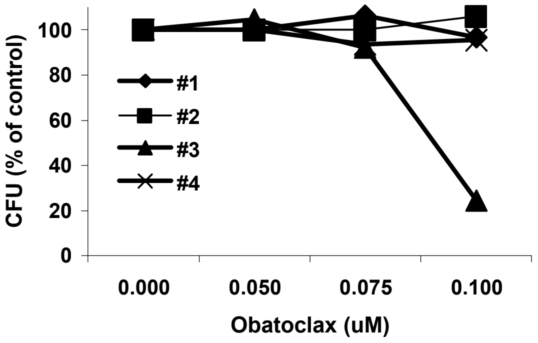

Single agent trials of obatoclax have been completed utilizing a variety of intravenous (IV) schedules. In studies utilizing short infusion durations (1-3 hours), dose-limiting toxicities (DLTs) have been infusion-associated somnolence, ataxia and euphoria. These toxicities are less frequent and less severe when obatoclax is administered by a 3-hour infusion, and even less frequent and less severe when obatoclax is administered utilizing a 24-hour infusion.

In vitro studies have suggested that prolonged exposure lowers the dose of obatoclax needed for inhibition of cell growth, but clinical data comparing the efficacy of the 3-hour and 24-hour infusion schedules are not available. Although the concentration that caused 50% inhibition (IC50) for C33A cervical cancer cells using a 3-hour infusion was 0.28 µM, the IC50 for the same cell line using a 24-hour exposure was 0.075 µM. In patients the peak plasma concentration with a 20 mg/m^2 3-hour obatoclax infusion was 99.7 ng/mL, while the peak plasma concentration reached utilizing a 28 mg/m^2 24-hour infusion was 10.8 ng/mL. Thus the peak plasma concentration ratio of a 24-hour MTD infusion to that of a 3-hour MTD infusion was 0.11 (10.8/99.7), while the IC50 ratio for cells exposed to 24-hours of obatoclax compared to 3 hours of obatoclax was 0.27 (0.075/0.28). Although the IC50 values decrease as exposure is prolonged to 24 hours, the peak plasma concentrations at MTD doses decrease even more, indicating that clinical trials are needed to provide information on the relationship of efficacy to the schedule of obatoclax.

Obatoclax has displayed single-agent antitumor activity in all 3 clinical Phase I trials including in patients with myelodysplastic syndromes, refractory chronic lymphocytic leukemia, and non-Hodgkin's lymphoma. There was also dramatic evidence of benefit in 1 patient with AML. A 70-year-old woman with a prior history of adjuvant anthracycline chemotherapy for breast cancer later developed AML with an 11q23 translocation. She was treated with obatoclax 20 mg/m^2 over 24 hours and achieved a cytogenetic CR with complete hematological recovery 8 days after her first infusion, with no significant toxicities. This CR was maintained for over 8 months during which obatoclax treatment was continued. When the patient relapsed, additional treatment with obatoclax at a dose of 28 mg/m^2 over 24 hours for 4 days in a row was ineffective.

The current study is designed to fully evaluate the therapeutic potential of obatoclax in older patients with previously untreated AML as an alternative to initial standard induction chemotherapy. The study is also designed to compare the efficacy and safety profiles of obatoclax administered for 3 consecutive days by 3-hour or 24-hour infusion in a pilot schedule evaluation in order to determine which schedule of obatoclax to utilize in the Phase II portion of the study.

4.1.1. Summary of Potential Risks to Human Subjects and Guidance to the Investigator

4.1.1.1. General Handling of Obatoclax

Obatoclax must be administered only through a free-flowing, non-polyurethane-containing peripheral IV site, or central venous catheter or access device. Obatoclax is a dye, and thus small amounts of obatoclax on bandages, clothing or other items are easily seen. However, obatoclax is not considered to be toxic, and thus items stained with obatoclax can be disposed of in regular

trash. Since solutions may leach through silicone catheters, obatoclax may stain items that come in contact with the outside of the catheter. Obatoclax is not an irritant to skin and is not genotoxic. However, teratogenicity testing has not been performed with obatoclax, and thus pregnant women should not receive obatoclax or be in direct contact with obatoclax or with obatoclax-stained items. Materials used for the medical delivery of obatoclax, such as needles, syringes, and catheter tubing, should be disposed of as medical waste in appropriate containers.

4.1.1.2. Nervous System Adverse Events

A range of neurologic and psychiatric symptoms have occurred during and shortly after obatoclax infusion, as documented in the adverse event (AE) listings and in the Adverse Events of Special Interest section of the Investigator's Brochure. Commonly occurring neurologic and psychiatric symptoms have included somnolence and euphoric mood. Data to date support a relationship between the infusion duration of obatoclax and the occurrence of such symptoms, with both the frequency and severity of neurologic and psychiatric symptoms decreasing as the infusion duration is increased from 1 hour to 24 hours. In the event of Grade 3 central nervous system (CNS) events, patients have been successfully re-dosed following a dose reduction. Although the CNS symptoms typically resolve within hours, persistent symptoms have responded to a temporary (1 week) interruption of treatment.

Patients should be monitored carefully during and shortly after an infusion of obatoclax. Any persistent neurological or psychiatric toxicity of ≥Grade 2 should be promptly evaluated using appropriate physical examination and imaging modalities. During and shortly after obatoclax IV infusions, patients should be careful standing up and walking, as confusional states and ataxia can occur. Patients must be accompanied home from the clinic after receiving an obatoclax infusion or after being started on a prolonged obatoclax infusion. Patients should not operate a motor vehicle or other potentially dangerous equipment during or shortly after an infusion of obatoclax.

4.1.1.3. Respiratory System

Chest radiographs and pulmonary function tests must be performed at baseline, at intervals specified in Section 6.2, and at the 28-day post last dose visit.

4.1.1.4. Cytokine Release Syndrome

Approximately 5% of patients have experienced a cytokine release syndrome during the infusion of obatoclax. This syndrome includes hypersensitivity symptoms as well as additional symptoms such as hypertension and rigors. In order to prevent the cytokine release syndrome, patients should receive H1 and H2 blockers such as diphenhydramine and famotidine prior to the start of obatoclax infusions. If a cytokine release syndrome occurs, the obatoclax infusion should be stopped immediately, and the patient treated symptomatically with additional diphenhydramine and famotidine, or with acetaminophen or meperidine for rigors.

5. TRIAL OBJECTIVES AND PURPOSE

5.1. Primary objective

- Determine the morphologic CR rate of obatoclax single-agent therapy in older patients with previously untreated AML.

5.2. Secondary objectives

- Based on the safety and efficacy profile of obatoclax in the pilot schedule evaluation, determine whether 3-hour or 24-hour obatoclax infusions should be utilized in the Phase II portion of the trial.
- Describe the duration of CR following treatment with obatoclax.
- Describe the safety profile of obatoclax administered every 2 weeks to older patients with previously untreated AML.
- Describe the mortality rate within 30 days of the first dose of obatoclax.
- Describe the utilization of red blood cell and platelet transfusions following the start of obatoclax.
- Characterize the pharmacokinetic (PK) profile of obatoclax administered every 2 weeks to older patients with previously untreated AML.
- Characterize baseline expression of bcl-2 family members in leukemic cells to investigate the hypothesis that increased expression of specific pro-survival family members or decreased expression of bax and bak correlates with response to obatoclax.

6. INVESTIGATIONAL PLAN

6.1. Overall Study Design and Plan: Description

This is a multicenter, open-label, 2-stage Phase II study of single-agent obatoclax mesylate administered for 3 consecutive days every 2 weeks in older patients with previously untreated AML. The 2-stage Phase II portion of the study is preceded by a pilot safety evaluation and a pilot schedule evaluation. Since the schedules for obatoclax administration evaluated previously do not include administering obatoclax by 3-hour infusion for 3 consecutive days, the pilot safety evaluation will first be conducted to evaluate the safety parameters of this schedule at a stepped-down dose in 3 patients. Then, the pilot schedule evaluation will be conducted to select the schedule to be evaluated in the Phase II portion of the trial (3-hour vs. 24-hour infusion; each administered for 3 consecutive days). The study design logic diagram is illustrated in Appendix 1, section 19.1.

During the first cycle of treatment (Cycle 1, Days 1-3), samples for PK analysis will be collected at appropriate time points throughout and beyond the infusional period. Therefore, overnight hospitalization will be required. Subsequent infusions may be administered on an outpatient basis. No investigational or commercial agents or therapies other than those described herein may be administered with the intent to treat the patient's malignancy.

Table 4 displays the timing of all study assessments and procedures. Baseline evaluations are to be conducted within 7 days prior to the start of obatoclax administration. Disease assessments including a unilateral bone marrow aspirate and biopsy must be done within 42 days prior to the start of therapy. Unless stated otherwise, all clinical assessments on Day 1 of treatment are to be performed prior to the patient receiving treatment.

6.1.1. Pilot Safety Evaluation

For the pilot safety evaluation, the first 3 patients enrolled will receive a 3-hour infusion utilizing the stepped-down dose of 30 mg/day for 3 consecutive days (total dose of 90 mg over 3 days). During Cycle 1 of this pilot safety evaluation:

- If ≤1 of the 3 patients has a DLT, 45 mg/day for 3 consecutive days (total dose of 135 mg over 3 days) will be utilized for the 3-hour infusions in the subsequent pilot schedule evaluation.
- If ≥2 of the 3 patients have DLTs, an additional pilot safety evaluation dosage group will be enrolled to receive 20 mg/day as a 3-hour infusion for 3 consecutive days (total dose of 60 mg over 3 days).

Similar rules will apply to the 20 mg/day as a 3-hour infusion for 3 consecutive days dosage group if utilized in the pilot safety evaluation:

- If ≤1 of the 3 patients has a DLT, 20 mg/day as a 3-hour infusion for 3 consecutive days (total dose of 60 mg over 3 days) will be utilized for the 3-hour infusions in the pilot schedule evaluation.
- If ≥2 of the 3 patients have DLTs, the 3-hour infusion schedule will not be utilized further, and the pilot schedule evaluation will utilize only the 24-hour schedule for obatoclax administration.

6.1.2. Pilot Schedule Evaluation

In the pilot schedule evaluation, 16 patients will be randomized to treatment with 8 patients assigned to the group receiving 45 mg/day obatoclax administered as a 3-hour infusion for 3 consecutive days (total dose 135 mg over 3 days; dependent on the results of the pilot safety evaluation) and 8 patients assigned to the group receiving 60 mg/day obatoclax administered as a continuous infusion for 3 consecutive days (total dose 180 mg over 3 days), each to be repeated 2 weeks later as an initial induction therapy.

The first 3 patients enrolled into the pilot schedule evaluation receiving 45 mg/day over 3 hours will be evaluated for safety after Cycle 1. If ≤1 of these 3 patients has a DLT, then 45 mg/day will continue to be utilized for this arm of the pilot schedule evaluation with dose reductions for toxicities as needed. If ≥2 patients have DLTs during Cycle 1, then 30 mg/day will be utilized for the remainder of this arm of the pilot schedule evaluation portion of the trial. In this case, 8 additional patients would be enrolled into this arm.

If no CRs are observed in either the 3-hour or the 24-hour continuous infusion group, the trial will stop. If a CR is observed in at least 1 of the 8 patients in either group, the safety and efficacy profiles of the 3-hour and 24–hour infusion groups will be compared to determine which schedule of obatoclax administration to utilize in the Phase II portion of the trial.

6.1.3. Phase II Portion of the Study

The Phase II portion of the trial with a formal 2-stage design will enroll 37 patients into Stage 1 using the more favorable obatoclax administration schedule from the pilot schedule evaluation. If ≥3 of the 37 patients enrolled achieve a CR by the end of Stage 1, the trial will proceed to Stage 2, which will enroll an additional 47 patients for a total of up to 84 patients.

Patients achieving a CR (documented by a repeat bone marrow examination on Day 28 or earlier) in any portion of this trial will receive 4 additional cycles of obatoclax as consolidation. Thus, patients achieving CR will receive a total of 6 cycles of obatoclax treatment. Patients who fail to achieve a CR following induction therapy (ie, Cycles 1 and 2) will be removed from study.

Table 4: Schedule of Study Assessments

	Baseline [b]	Cycles 1-6 [a] Day 1 [c]	Cycles 1-6 [a] Day 2	Cycles 1-6 [a] Day 3	Cycles 1-6 [a] Day 8	28 Days After Last Dose
Obatoclax (GX15-070MS) administration [d]		X	X	X		
Informed consent	X					
Demographics	X					
Medical history	X					
Concomitant medications	X---X					
Physical examination (including neurological examination)	X	X			X	X
Vital signs	X	X			X	X
Height	X					
Weight	X	X			X	X
Performance status	X	X			X	X
CBC with differential, platelets [e]	X	X			X	X
Serum chemistry [f]	X	X			X	X
Urinalysis [g]	X					X
ECG [h]	X			X [i]		X
Adverse event evaluation	X---X					
Unilateral bone marrow aspirate & biopsy [j]	X	Repeated as clinically indicated				
Chest radiograph	X					X
Pulmonary function tests	X					X
Pharmacokinetics		X--------At specified times [k] during Cycle 1 only--------X				

Table 4: Schedule of Study Assessments (continued)

a Obatoclax will be administered as a 3-hour infusion for 3 consecutive days or as a continuous infusion for 3 consecutive days to be repeated 2 weeks later as an initial induction therapy. Patients achieving a CR will then receive 4 additional 3-day cycles every 2 weeks as consolidation. Patients who fail to achieve a CR following the initial induction therapy will be removed from study.

b Baseline evaluations will be conducted within 7 days prior to the start of obatoclax administration. Unilateral bone marrow aspirate and biopsy must be done within 42 days prior to the start of therapy.

c All clinical assessments on Day 1 of treatment are to be performed prior to receiving treatment.

d Obatoclax will be administered either as a 3-hour infusion for 3 consecutive days or as a continuous infusion for 3 consecutive days. The infusion bag of obatoclax will be changed for each 24-hour period.

e To include absolute neutrophil count, lymphocytes, monocytes, eosinophils, and basophils. CBCs to be obtained on Day 1 of each cycle, every 2-3 days for the first week of Cycle 1, and on Day 8 of each cycle.

f Albumin, alkaline phosphatase, total bilirubin, bicarbonate, blood urea nitrogen, calcium, chloride, creatinine, glucose, lactic dehydrogenase, magnesium, phosphorus, potassium, total protein, aspartate transaminase (AST [SGOT]), alanine transaminase (ALT [SGPT]), sodium, and uric acid.

g Urinalysis to include dipstick (urine pH, glucose, protein, blood, ketones) and microscopy.

h Repeated as clinically indicated.

i On Day 3 of Cycle 1 only, ECG to be performed 30 minutes before the end of the infusion.

j To be conducted at baseline and on Day 28 (unless a CR has been confirmed earlier), after obatoclax consolidation therapy, and as clinically indicated. Bone marrow samples will be evaluated for morphology and cytogenetics, as indicated. All bone marrow aspirate and biopsy samples collected at baseline and during the study will be reviewed by an independent central pathologist.

k For all patients receiving 3-hour obatoclax infusions, blood samples will be drawn at the following points, during Cycle 1 only: on Day 1 timed from the start of the infusion: predose, 0.5, 1, 2.5, 3 (prior to end of infusion); on Day 1 timed from the end of the infusion: 0.5, 1, 3, and 9 hours, on Day 2: predose and 2.5 hours (30 minutes prior to end of infusion); on Day 3 timed from the start of the infusion: predose, 0.5, 1, 2.5, 3 (prior to end of infusion); and on Day 3 timed from the end of the infusion: 0.5, 1, 3, 9, and 21 hours, with n=21 samples over 3 days. For all patients receiving 72-hour obatoclax infusions, 14 blood samples will be collected during Cycle 1 only. Blood samples will be drawn at: pre-dose, 1, 4, 8, 22, 32, 46, 56, and 70 hours (timed from the start of the infusion). Following termination of the 72-hour infusion period of Cycle 1, additional samples will be drawn at 0.5, 1, 3, 9, and 21 hours, timed from the end of the infusion.

6.2. Study Assessments

6.2.1. Demographic and Baseline Assessments

The following data will be collected at baseline: patient initials, date of birth, sex, race, ethnicity, previous history of AML, general previous medical history, baseline malignant disease-related symptoms, baseline concomitant medications, physical examination including neurological examination, vital signs, height, weight, performance status, complete blood count (CBC), serum chemistry, urinalysis, electrocardiogram (ECG), pulmonary function tests, disease assessments including a unilateral bone marrow aspirate and biopsy, and chest radiograph.

6.2.2. Efficacy Assessments

Clinical response will be evaluated according to standard criteria as described in Section 10.1. The following assessments will be conducted:

- Peripheral blood counts (at baseline and monitored throughout the study)
- Bone marrow aspirates and biopsies (at baseline and on Day 28, unless a CR has been confirmed earlier, after obatoclax consolidation therapy, and as clinically indicated)
- Bone marrow cytogenetics (at baseline, repeated in case of CR and as clinically indicated)

All bone marrow aspirate and biopsy samples collected at baseline and during the study will be reviewed by an independent central pathologist. Shipping information will be provided in the Lab Reference Manual. The evaluation of the independent central pathologist will not be utilized for clinical decisions during the trial.

6.2.3. Safety Assessments

Patients will be regularly monitored throughout the study for emerging AEs as defined in Section 11. In addition, the following safety assessments will be conducted:

- Emerging AEs (monitored throughout the study)
- Physical and neurological examinations (at baseline and on Days 1 and 8 of each cycle)
- Vital signs (at baseline, on Days 1 and 8 of each cycle)
- Body weight (at baseline and on Days 1 and 8 of each cycle)
- Performance status (at baseline and on Days 1 and 8 of each cycle)
- Complete blood count (at baseline, on Day 1 of each cycle, every 2 to 3 days for the first week of Cycle 1, and Day 8 of each cycle)
- Serum chemistry (at baseline and on Days 1 and 8 of each cycle)
- Urinalysis (at baseline and at the 28 day follow-up visit)

- Electrocardiogram ([ECG] at baseline, 30 minutes before the end of infusion on Day 3 in Cycle 1, and at the 28 day follow-up visit, to be repeated as clinically indicated)
- Chest radiographs (at baseline and at the 28 day follow-up visit)
- Pulmonary function tests (at baseline and 28 day follow-up visit)

6.2.3.1. Pregnancy

The investigator, or his/her designee, will collect pregnancy information on any male patient's female partner who becomes pregnant while the patient is participating in this study. The investigator, or his/her designee, will record pregnancy information on the appropriate form and submit it to Gemin X, Inc. (hereafter referred to as Gemin X) or its designee within 2 weeks of learning of a pregnancy. The outcome of the pregnancy will be determined through follow up. Information on the status of the mother and child will be forwarded to Gemin X or its designee. Generally, follow up will be no longer than 6 to 8 weeks following the estimated delivery date. Any premature termination of the pregnancy will be reported.

While pregnancy itself is not considered to be an AE or SAE, any pregnancy complication or elective termination of a pregnancy for medical reasons will be recorded as an AE or SAE, as described in Section 11.1 and will be followed as described in Section 11.6.

A spontaneous abortion is always considered to be an SAE and will be reported as described in Section 11.5.3. Furthermore, any SAE occurring as a result of a post-study pregnancy and considered reasonably related to the investigational product by the investigator, will be reported to Gemin X or its designee as described in Section 11.6.1. While the investigator is not obligated to actively seek this information in former study participants, he/she may learn of an SAE through spontaneous reporting.

6.2.4. Translational Medicine Studies

Samples of leukemic cells from baseline peripheral blood samples or bone marrow aspirates will be used in translational medicine studies at a central laboratory. These studies will characterize the baseline protein and/or mRNA expression of bcl-2 family members in leukemic cells to investigate the hypothesis that increased expression of specific pro-survival family members or decreased expression of bax and bak correlates with response to obatoclax. Shipping information will be provided in the Lab Reference Manual.

7. SELECTION AND WITHDRAWAL OF SUBJECTS

To assess any potential impact on patient eligibility with regard to safety, the investigator must refer to the Investigator's Brochure (IB) for detailed information regarding warnings, precautions, contraindications, AEs, and other significant data pertaining to the investigational product being used in this study.

7.1. Subject Inclusion Criteria

Patients will be considered eligible for inclusion in this study only if all of the following criteria apply:

1. Patients must have histologically or cytologically confirmed AML.
2. Patient must not have received prior chemotherapy for AML with the exception that the patients enrolled in the pilot safety evaluation may have had 1 prior therapy
3. Patients must be ≥70 years of age.
4. Patients must have an Eastern Cooperative Oncology Group (ECOG) Performance Status ≤2 (see Appendix 2, Section 19.2).
5. Patients must have normal organ function as defined below:
   - Total bilirubin ≤2 mg/dL unless resulting from hemolysis,
   - Aspartate transaminase (AST [SGOT])/alanine transaminase (ALT [SGPT]) ≤2.5 x institutional upper limit of normal,
   - Creatinine within normal institutional limits

     OR

     Creatinine clearance ≥50 mL/min/1.73 m^2 for patients with creatinine levels above institutional normal.
6. Because of the potential for unknown risks to an unborn child that could result from exposure to obatoclax, men with partners of child-bearing potential and women participating in the study must agree to not participate in sexual activities that could result in pregnancy for the duration of study participation.
7. Patients must be willing to submit to blood sampling for the planned PK analyses.
8. Patients must have the ability to understand and the willingness to sign a written informed consent form.

7.2. Subject Exclusion Criteria

Patients are excluded from this study if any of the following criteria apply:

1. Patients who have received or are receiving any other investigational or commercial agents or therapies administered with the intent to treat their malignancy, or patients who have had prior exposure to obatoclax (with the exception that the patients enrolled in the pilot safety evaluation may have had 1 prior therapy)

2. Patients with a history of allergic reactions attributed to components of the formulated product (PEG300 and polysorbate 20) are excluded from this study.
3. Patients with a history of seizure disorders or CNS leukemia are excluded from this study.
4. Patients with uncontrolled, intercurrent illness including, but not limited to, symptomatic neurological illness; active, uncontrolled, systemic infection considered opportunistic, life-threatening, or clinically significant at the time of treatment; symptomatic congestive heart failure; unstable angina pectoris; cardiac arrhythmia; significant pulmonary disease or hypoxia; or psychiatric illness/social situations that would limit compliance with study requirements are excluded from this study.
5. Human immunodeficiency virus (HIV)-positive patients receiving combination anti-retroviral therapy are excluded from this study.

7.3. Subject Withdrawal Criteria

Patients who fail to achieve a CR following the initial induction therapy (Cycles 1 and 2) will be removed from study. In addition, a patient may be withdrawn from treatment with obatoclax because of AEs, investigator or patient refusal to continue, disease progression, or death. If a patient is withdrawn from treatment with obatoclax for any reason other than death, all planned evaluations, including those at the visit at the 28 day follow-up visit, must be completed. Patients withdrawing from the study will not be replaced.

A patient may withdraw from the study at anytime. However, should such an event occur, the patient should be encouraged to complete any safety evaluations expected at that time and to comply with the safety evaluations of the 28 day follow-up visit.

A patient will be deemed to have completed the study after having received the initial induction therapy (Cycles 1 and 2) of obatoclax therapy and, if appropriate, 4 additional cycles as consolidation.

8. TREATMENT OF SUBJECTS

8.1. Description of Study Drug

8.1.1. Definition of Dose-Limiting Toxicity

In human Phase I trials using 1-hour, 3-hour, or 24-hour infusions, infusion-related neurological AEs have been observed (see IB for details) and are to be considered DLTs when of ≥Grade 3. Grade 3 or 4 non-hematological toxicity not ameliorated by symptomatic-directed therapy is also defined as a DLT.

Management and dose modifications associated with the above AEs are outlined in Section 8.1.3.

8.1.2. Obatoclax Administration

8.1.2.1. Pilot Safety Evaluation

For the pilot safety evaluation, the first 3 patients enrolled will receive a 3-hour infusion utilizing the stepped-down dose of 30 mg/day for 3 consecutive days (total dose of 90 mg over 3 days). During Cycle 1 of this pilot safety evaluation:

- If ≤1 of the 3 patients has a DLT, 45 mg/day for 3 consecutive days (total dose of 135 mg over 3 days) will be utilized for the 3-hour infusions in the subsequent pilot schedule evaluation.
- If ≥2 of the 3 patients have DLTs, an additional pilot safety evaluation dosage group will be enrolled to receive 20 mg/day as a 3-hour infusion for 3 consecutive days (total dose of 60 mg over 3 days).

Similar rules will apply to the 20 mg/day as a 3-hour infusion for 3 consecutive days dosage group if utilized in the pilot safety evaluation:

- If ≤1 of the 3 patients has a DLT, 20 mg/day as a 3-hour infusion for 3 consecutive days (total dose of 60 mg over 3 days) will be utilized for the 3-hour infusions in the pilot schedule evaluation.
- If ≥2 of the 3 patients have DLTs, the 3-hour infusion schedule will not be utilized further, and the pilot schedule evaluation will utilize only the 24-hour schedule for obatoclax administration.

8.1.2.2. Pilot Schedule Evaluation

In the pilot schedule evaluation, 16 patients will be randomized to treatment with either 45 mg/day obatoclax administered as a 3-hour infusion for 3 consecutive days (total dose 135 mg over 3 days; dependent on the results of the pilot safety evaluation) or 60 mg/day obatoclax administered as a continuous infusion for 3 consecutive days (total dose 180 mg over 3 days), each to be repeated 2 weeks later as an initial induction therapy.

The first 3 patients enrolled into the pilot schedule evaluation receiving 45 mg/day over 3 hours will be evaluated for safety after Cycle 1. If ≤1 of these 3 patients has a DLT, then 45 mg/day will continue to be utilized for this arm of the pilot schedule evaluation with dose reductions for toxicities as needed. If ≥2 patients have DLTs during Cycle 1, then 30 mg/day will be utilized for the remainder of this arm of the pilot schedule evaluation portion of the trial. In this case, 8 additional patients would be enrolled into this arm.

If no CRs are observed in either the 3-hour or the 24-hour continuous infusion group, the trial will stop. If a CR is observed in at least 1 of the 8 patients in either group, the safety and efficacy profiles of the 3-hour and 24–hour infusion groups will be compared to determine which schedule of obatoclax administration to utilize in the Phase II portion of the trial.

8.1.2.3. Phase 2 Portion of the Study

Obatoclax will be administered either as a 45 mg flat dose (depending on results from the pilot safety evaluation) over 3 hours for 3 consecutive days (for a total of 135 mg over 3 days) or as a flat dose of 60 mg over 24 hours for 3 consecutive days (for a total of 180 mg over 3 days) to be repeated 2 weeks later as an initial induction therapy (ie, Cycles 1 and 2). When obatoclax is administered by continuous infusion, the infusion bag will be changed for each 24-hour period as applicable.

8.1.3. Dosing Delays/Dose Modifications

If a patient experiences a DLT, treatment must be modified. Once the DLT has resolved to <Grade 3, the patient may resume treatment with a reduction to a flat dose of 30 mg/day for the 3-hour 45 mg/day infusion regimen, 20 mg/day for the 3-hour 30 mg/day infusion regimen, or 45 mg/day for the 24-hour 60 mg/day infusion regimen. If DLTs recur following the dose reduction, the patient will be removed from the study.

If treatment is delayed for >28 days from the date of the last dose received, the patient should be removed from the study.

There will be no dose adjustment for myelosuppression.

8.1.4. Supportive Care Guidelines

A cytokine-release syndrome has been reported during infusion of obatoclax. Prophylaxis with H-1 and H-2 blockers is recommended prior to each cycle. Baseline electrolyte abnormalities should be corrected prior to treatment initiation. If nausea or vomiting emerges as an acute toxicity, anti-emetic prophylaxis will be instituted as appropriate. Full supportive care measures will be offered to treat any emerging DLTs.

Supportive care measures including those directed at controlling symptoms resulting from hematological malignancies (blood products, antibiotics, IV immunoglobulins, hematopoietic growth factors, etc.) are allowed.

8.1.5. Accidental Overdose

There are no known antidotes to obatoclax. Accidental overdoses should be treated with general supportive care measures.

8.1.6. Duration of Therapy

If a patient achieves a CR after the initial 2 cycles of induction therapy (Cycles 1 and 2), that patient will receive 4 additional cycles of consolidation given 2 weeks apart (Cycles 3-6). Thus, patients achieving CR will receive a total of 6 cycles of obatoclax treatment.

Patients who fail to achieve a CR following the initial induction therapy (Cycles 1 and 2) will be removed from study.

8.2. Concomitant Medications

All concomitant medications taken during the study will be recorded in the case report form (CRF) with indication and dates of administration.

Supportive care measures, including those directed at controlling symptoms resulting from hematological malignancies (blood products, antibiotics, IV immunoglobulins, hematopoietic growth factors, etc.) are allowed. (See also Section 8.1.4). However, any other treatment for the underlying malignant disease, with the exception of hydroxyurea, is prohibited, including other experimental therapies.

8.3. Treatment Compliance

Study treatment will be administered under the direction of the investigator at the study site. At the time of dosing, the actual dose administered will be recorded in the CRF.

8.4. Randomization and Blinding

This is an open-label study in which study medication will not be blinded. Central randomization will be performed for assignment of treatment arms in the Pilot Schedule portion of the study.

8.5. Follow Up After Completion of Obatoclax Therapy or Withdrawal from Study

All patients completing obatoclax therapy or who have withdrawn from the study will be followed for date of progression after obatoclax therapy and date of death. Follow up information will be obtained every 3 months.

9. STUDY DRUG MATERIALS AND MANAGEMENT

9.1. Study Drug

All study medication will be provided by the Sponsor as open-label stock as GX15-070MS (designation number for obatoclax) as a lyophilized cake for reconstitution and dilution. Inactive ingredients include polyethylene glycol (PEG) 300 and polysorbate 20.

9.2. Study Drug Packaging and Labeling

Clinical-trial supplies will be provided in cartons containing 2 vials:

- Lyophilized GX15-070MS Formulation (30 mg single-use vial) containing the active ingredient GX15-070MS (30 mg)
- Diluent for GX15-070MS (40 mL single-use vial): 96.2% PEG 300 (38.48 mL) and 3.8% Polysorbate 20 (1.52 mL)

9.3. Study Drug Storage

The vials must be refrigerated at 2-8°C (36-46°F) in the supplied carton. DO NOT FREEZE. Study drug storage and handling is described further in Section 9.7.

9.4. Study Drug Preparation

9.4.1. Incompatibilities

The compatibility of obatoclax with solutions other than 5% Dextrose for Injection, United States Pharmacopeia (USP) containing ≥9.62% PEG 300 and ≥0.38% polysorbate 20 has not been examined. The use of other solutions or additives is not allowed.

Obatoclax is not compatible with fluid paths or infusion sets containing di-2-(ethylhexyl) phthalate (DEHP), and therefore, material containing DEHP must not be used. The use of a soft non-DEHP fluid path, an infusion set with polyethylene-lined tubing, and a non-DEHP pump segment fitted with a 0.22 µm filter are required. Central or peripheral venous catheters with polyurethane components are not recommended to be used with obatoclax as the formulated drug product may not be compatible with the catheter. Certain catheters composed of silicone components can be used (See Investigator's Brochure for details).

9.4.2. Reconstitution

Note that intense, high-speed vortexing is required for complete dissolution of the lyophilized cake. Obatoclax will stain objects red; contact with non-disposable equipment should be avoided (See the material safety data sheet [MSDS] for cleaning procedure).

Using aseptic techniques, reconstitute obatoclax with the supplied diluent mixture and commercially-available 5% Dextrose for Injection, USP as follows:

1. Remove obatoclax, the supplied diluent, and commercially-available 5% Dextrose for Injection, USP from refrigeration at least 1 hour prior to preparation to allow all components to reach room temperature.
2. Aseptically remove caps and tabs of the plastic seals to expose rubber stoppers.
3. Clean stoppers with germicidal or alcohol swab.
4. Aseptically remove 2 mL of 5% Dextrose for Injection, USP using a sterile, single-use syringe and inject it slowly into the vial containing the obatoclax lyophilized cake.
5. Agitate the vial vigorously using a vortex shaker at high speed for at least 1 min to create a fine dispersion.
6. Aseptically remove 8 mL of diluent using a sterile, single-use syringe and inject it slowly into the vial containing the obatoclax dispersed in 5% Dextrose for Injection, USP.
7. Agitate the vial vigorously using a vortex shaker at high speed for at least 2 min or longer until the drug product is completely dissolved. Each reconstituted vial now contains 30 mg obatoclax to a reconstituted concentration of 3 mg obatoclax per mL.

   Note: It is preferable to let the reconstituted vial stand inverted for at least 10 min to allow bubbles to evacuate the solution away from the syringe insertion site. Shining light across the sample can help visualize particles.
8. Inspect solution for dark particulate matter after reconstitution.

9.4.3. Dilution

Three constituents are to be added to a non-DEHP fluid path container, in the following order:

1. 5% Dextrose for Injection, USP (V_{D5W})
2. Diluent (96.2% PEG 300, 3.8% Polysorbate 20; $V_{diluent}$), if required, to ensure a final concentration of 11.544% PEG 300 and 0.456% polysorbate 20.
3. Reconstituted obatoclax ($V_{obatoclax}$), containing 3 mg/mL obatoclax in 1.0% Dextrose for Injection, USP with 76.96% PEG 300 and 3.04% polysorbate 20.

Dose reconstitution is presented in Table 5 for the 30 mg, 45 mg, and 60 mg doses to be used in the trial, and the planned dose reduction to 20 mg in the event of DLT.

Table 5: Dose Reconstitution

Total Dose of Obatoclax (mg)	Reconstituted Obatoclax ($V_{obatoclax}$; mL)	Diluent ($V_{diluent}$; mL)	5% Dextrose for Injection, USP (V_{D5W}; mL)
20	6.7	24.7	218.6
30	10	22	218
45	15	18	217
60	20	14	216

D5W = 5% dextrose in sterile water; mg = milligram; mL = milliliter; % = percentage; USP = United States Pharmacopeia; V = volume.

The following details a step-by-step procedure to be followed for dilution. Note that the 5% Dextrose for Injection, USP is not provided.

1. Clean stoppers with germicidal or alcohol swab.
2. Aseptically transfer the appropriate volume of 5% Dextrose for Injection, USP (V_{D5W}) into the non-DEHP container.
3. Aseptically transfer the appropriate volume of diluent ($V_{diluent}$) using a sterile, single-use syringe and inject it slowly into the non-DEHP container.
4. Mix the solution by inverting the bag gently 5 to 10 times.
5. Finally, aseptically transfer the desired volume of reconstituted obatoclax ($V_{obatoclax}$) corresponding to the prescribed dose using a sterile, single-use syringe and inject it slowly into the non-DEHP container.
6. Mix the solution by inverting the bag gently 5 to 10 times.
7. The total volume to be infused is 250 mL. Follow the manufacturer’s instructions for the transfer of fluid into the fluid path container.

9.5. Infusion Administration

The study drug should be administered by infusion using an infusion set with polyethylene-lined tubing as described in the following steps.

1. Close the blue clamp/valve.
2. Clean stoppers with germicidal or alcohol swab.
3. Connect the infusion set to the 250 mL container containing reconstituted and diluted drug.
4. For the 3-hour infusion, set the flow rate to deliver the dose volume of 250 mL over 3 hours (180 minutes) (~1.39 mL/min). For the 24-hour infusion, set the flow rate to deliver the dose volume of 250 mL over 24 hours (1,440 min) (~0.17 mL/min).

5. Infuse through a 0.22-micron filter into a free-flowing, peripheral vein or compatible central-venous catheter or access device.

Note: Follow the infusion set manufacturer's instructions for the infusion administration.

CAUTION: Do not refrigerate the reconstituted or diluted drug product. Parenteral product should be inspected visually for particulate matter and discoloration prior to administration whenever solution and container permit. Do not use if cloudiness or precipitate is observed.

9.6. Study Drug Accountability

The investigator is responsible for investigational-product accountability, reconciliation, and record maintenance. In accordance with all applicable regulatory requirements, the investigator or designated site staff must maintain investigational product-accountability records throughout the course of the study. This person(s) will document the amount of investigational product received from Gemin X and the amount administered to patients.

9.7. Study Drug Handling and Disposal

Investigational product must be dispensed or administered according to procedures described herein. Other instructions include:

- Store refrigerated at 2-8°C (36-46°F).
- Store in the original cardboard packaging.
- Do not freeze.
- Allow product to reach room temperature before reconstituting and diluting. An unopened vial of obatoclax may be at room temperature for up to 2 days prior to use.
- Reconstituted vials of obatoclax must be kept at room temperature and used within 72 hours.
- Infusion solutions of obatoclax must be kept at room temperature and used within 36 hours.
- Any unused drug remaining after reconstitution and after infusion must be discarded; see MSDS for safety information and disposal procedure.

Only patients enrolled in the study may receive investigational product, in accordance with all applicable regulatory requirements. Only authorized site staff may supply or administer investigational product. All investigational products must be stored in a secure area with access limited to the investigator and authorized site staff and under physical conditions that are consistent with investigational product-specific requirements.

Precaution should be taken to avoid direct contact with the investigational product. An MSDS describing occupational hazards and recommended handling precautions is available to the investigator.

10. ASSESSMENT OF EFFICACY

Patients will be assessed by standard criteria. Peripheral blood counts will be monitored throughout this study, and bone marrow aspirates and biopsies will be repeated as indicated.

10.1. Response Criteria

The criteria summarized in Table 6 have been used to assess response in AML studies [Cheson, 2003]. The primary endpoint of this trial will be morphologic CR. Cytogenetic CR will also be evaluated for those patients with abnormal cytogenetic results at baseline.

Table 6: Response Criteria in Acute Myeloid Leukemia

Criterion	Neutrophils (µL)	Platelets (µL)	Bone Marrow Blasts (%)	Other
Early treatment assessment [a]	NA	NA	<5	
Morphologic leukemia-free state	NA	NA	<5	Flow cytometry EMD absent
Morphologic CR	>1000	>100,000	<5	Transfusion EMD absent
Cytogenetic CR	>1000	>100,000	<5	Cytogenics normal, EMD absent
Molecular CR	>1000	>100,000	<5	Molecular negative, EMD absent
Partial remission	>1000	>100,000	>50 or decrease to 5-25	Blasts <5% if Auer rod positive

a Assessed 7-10 days after treatment
µL = microliter; CR = complete remission; EMD = extramedullary disease.

10.2. Recurrence Criteria

Following CR, relapse is defined as a reappearance of leukemic blasts in the peripheral blood or ≥5% blasts in the bone marrow not attributable to any other cause (e.g., bone marrow regeneration after consolidation therapy), appearance of new dysplastic changes, or reappearance or development of cytologically proven extramedullary disease [Cheson, 2003].

If there are no circulating blasts and the bone marrow contains 5%-20% blasts, a repeat bone marrow will be performed at least 1 week later to distinguish relapse from bone marrow regeneration, and the date of recurrence is defined as the date when >5% blasts were first observed in the marrow.

10.3. Duration of Response

The duration of CR is measured from the time criteria for CR are first met until the first date that recurrent disease is objectively documented. Remission-free survival is measured from the time

criteria for CR are first met until disease relapse or death due to any cause. Overall survival is measured from baseline until death due to any cause.

11. ASSESSMENT OF SAFETY

11.1. Safety Parameters

Safety will be assessed through the reporting of AEs and monitoring of physical and neurological examinations, vital signs, body weight, performance status, CBC, serum chemistries, urinalysis, EKGs, and chest radiographs (See also Section 6.2.3).

11.1.1. Definitions

An **adverse event** is defined as any untoward medical occurrence in a patient or clinical investigation subject, temporally associated with the use of a medicinal product, whether or not considered related to the medicinal product. Therefore, an AE can be any unfavorable and unintended sign (including a clinically-significant abnormal laboratory finding), symptom, or disease (new or exacerbated) temporally associated with the use of a medicinal product.

A **serious adverse event** is any untoward medical occurrence that, at any dose:

1. Results in death; NOTE: If an event leads to the death of a patient, the actual event that leads to death is the SAE; death is not to be listed as the SAE.
2. Is life-threatening; NOTE: The term "life-threatening" in the definition of "serious" refers to an event in which the patient was at risk of death at the time of the event. It does not refer to an event, which hypothetically might have caused death, if it were more severe.
3. Requires hospitalization or prolongation of existing hospitalization; NOTE: In general, hospitalization signifies that the patient has been detained (usually involving at least an overnight stay) at the hospital or emergency ward for observation and/or treatment that would not have been appropriate in the physician's office or out-patient setting. Complications that occur during hospitalization are AEs. If a complication prolongs hospitalization or fulfills any other serious criteria, the event is serious. When in doubt as to whether other events meet the serious criteria, the event is to be considered serious. When in doubt as to whether "hospitalization" occurred or was necessary, the AE should be considered serious. Hospitalization for elective treatment of a pre-existing condition that did not worsen from baseline is not considered an AE.
4. Results in significant disability/incapacity; NOTE: The term "significant disability" means a substantial disruption of a person's ability to conduct normal life functions. This definition is not intended to include experiences of relatively minor medical significance such as uncomplicated headache, nausea, vomiting, diarrhea, influenza, and accidental trauma (e.g., sprained ankle) which may interfere or prevent everyday life functions, but do not constitute a substantial disruption.
5. Is a congenital anomaly/birth defect.

Medical or scientific judgment should be exercised in deciding whether reporting is appropriate in other situations, such as important medical events that may not be immediately life-

threatening or result in death or hospitalization, but may jeopardize the patient or may require medical or surgical intervention to prevent 1 of the other outcomes listed in the above definition. These should also be considered serious. Examples of such events are invasive or malignant cancers, intensive treatment in an emergency room or at home for allergic bronchospasm, blood dyscrasias or convulsions that do not result in hospitalization, or development of drug dependency or drug abuse.

The following are examples of disease-related events or outcomes that **do not qualify** as SAEs:

1. Situations in which a patient is hospitalized for scheduled or elective surgery for a condition unattributable to the study drug or that was present prior to taking study medication.
2. Symptoms or minor concurrent events that occur during an SAE and are related to the ongoing SAE, but do not meet the SAE criteria. These should be captured as AEs.
3. An AE that is serious in intensity (i.e., the patient describes the event as “serious”), but the event itself does not meet the SAE criteria.

“Lack of efficacy” per se will not be reported as an AE. The signs and symptoms or clinical sequelae resulting from a lack of efficacy will be reported if they fulfill the AE or SAE definition/criteria (including clarifications).

Abnormal laboratory findings (e.g., clinical chemistry, hematology, urinalysis) or other abnormal assessments (e.g., physical examination, vital signs, EKG, etc.) that are judged by the investigator as clinically significant will be recorded as AEs or SAEs if they meet the definition of an AE, as described above.

Clinically-significant (associated with symptoms or requiring treatment) abnormal laboratory findings or other abnormal assessments that are detected during the study or are present at baseline and significantly worsen following the start of the study will be reported as AEs or SAEs. However, clinically-significant abnormal laboratory findings or other abnormal assessments that are associated with the disease being studied, unless judged by the investigator as more severe than expected for the patient’s condition, or that are present or detected at the start of the study and do not worsen, will not be reported as AEs or SAEs.

The adverse-event reporting period (for AEs and SAEs) for this study begins with the first dose of study drug (Day 1) and ends (and includes) 28 days following the last dose of study drug. Any AE, including those that lead to death, that occurs more than 28 days after the final dose of study drug for which the investigator assesses as possibly, probably, or definitely related to the investigational product, should also be reported as an AE or SAE.

At each study visit, the patient is to be questioned regarding any AEs. All AEs whether observed or elicited by the investigator, the study staff, or reported by the patient will be documented in the medical chart and recorded as an AE in the CRF. At that time, the investigator must assess whether the AE is considered drug related and determine whether to continue or discontinue the study drug. This process is to occur on a continual basis throughout the patient’s participation in the clinical trial.

All AEs should be followed until they are resolved or a clinically-stable endpoint is reached.

11.2. Relationship to Study Drug

Each AE should be classified as to its relationship to the study drug. An event for which sufficient information exists to indicate that the etiology is either related or unrelated should be measured against the following criteria:

- **Definitely Related** – An event that follows a reasonable temporal sequence from administration of the study drug; follows a known or expected response pattern to the suspected study drug; and that is confirmed by improvement on stopping or reducing the dosage of the study drug and reappearance of the reaction on repeated exposure unless the latter is considered to be medically unethical.
- **Probably Related** – An event that follows a reasonable temporal sequence from administration of the study drug; follows a known or expected response pattern to the suspected study drug; is confirmed by stopping or reducing the dosage of the study drug; and could not be reasonably explained by the known characteristics of the patient's clinical state.
- **Possibly Related** – An event that follows a reasonable temporal sequence from administration of the study drug; follows a known or expected response pattern to the suspected study drug; but could readily have been produced by a number of other factors.
- **Not Related** – A medical condition present before the administration of study drug unless the condition worsens or episodes increase in frequency after administration of the study drug; symptoms that are reasonably determined to be the normal course of the disease state (NOTE: Life-threatening complications from cancer are considered SAEs); an event for which sufficient information exists to indicate that the etiology is related to a concomitant medication; medical events (e.g., unrelated illness, accident, or trauma) that occur during the clinical trial for which sufficient information exists to indicate that the etiology is not related to the study drug; or symptoms from procedures such as surgery or radiation which are not a direct result of receiving study drug.

The investigator is obligated to assess the relationship between investigational product and the occurrence of each AE/SAE. The investigator will use clinical judgment to determine the relationship. Alternative causes, such as natural history of the underlying disease(s), concomitant therapies, other risk factors, and the temporal relationship of the event to the investigational product will be considered and investigated. The investigator will also consult the IB and/or any available product information for marketed products in the determination of his/her assessment.

There may be situations when an SAE has occurred, and the investigator has minimal information to include in the initial report to Gemin X or its designee. However, it is imperative that the investigator always make an assessment of causality for every event prior to transmission of the SAE Report Form to Gemin X or its designee as causality is the primary criteria for determining the reporting schedule of SAEs to regulatory authorities. If an insufficient amount of information is available at the time of the event to make an accurate assessment of causality, the investigator is encouraged to be conservative in his/her opinion. Any new findings that would change or affect the investigator's initial opinion of causality may be included as an amendment to the report along with the change of causality status at anytime.

11.3. Intensity of Adverse Events

The investigator will make an assessment of intensity for each AE and SAE reported during the study. The assessment will be based on the investigator's clinical judgment. The intensity of each event recorded in the CRF should be assigned using the grading features of the National Cancer Institute (NCI) Common Terminology Criteria for Adverse Events (CTCAE) in Section 19.2.

An AE that is assessed as severe should not be confused with an SAE. Severity is a category utilized for rating the intensity of an event; both AEs and SAEs can be assessed as severe. An event is defined as "serious" when it meets 1 of the pre-defined outcomes as described in Section 11.1.1.

11.4. Recording Adverse Events

When an AE/SAE occurs, it is the responsibility of the investigator to review all documentation (e.g., hospital progress notes, laboratory and diagnostic reports) relative to the event. The investigator will then record all relevant information regarding the event on the CRF. It is not acceptable for the investigator to send photocopies of the patient's medical records to Gemin X or its designee in lieu of completion of the appropriate AE pages. However, there may be instances when copies of medical records for certain cases are requested by Gemin X or its designee. In this instance, all patient identifiers will be blinded on the copies of the medical records prior to submission to Gemin X or its designee.

The investigator will attempt to establish a diagnosis of the event based on signs, symptoms, and/or other clinical information. In such cases, the diagnosis should be documented as the event (the actual AE/SAE) and not the individual signs/symptoms.

The course of events (both AEs and SAEs) is to be recorded accurately in the CRF. If the grade of a continuing AE changes, a stop date should be entered for the current event, and the new grade should be represented by a new adverse-event entry into the CRF with the appropriate information.

11.5. Reporting Adverse Events

The investigator is responsible for the detection and documentation of events meeting the criteria and definition of an AE or an SAE as provided in this protocol. During the study, when there is a safety evaluation, the investigator or site staff will be responsible for detecting AEs and SAEs and reporting them promptly to Gemin X or its designee.

11.5.1. Prompt Reporting of SAEs

SAEs will be reported promptly to Gemin X or its designee as described in Table 7 once the investigator determines that the event meets the criteria for an SAE.

Table 7: Timeframes for Reporting All Serious Adverse Events

	Timeframe	Document
Initial SAE Report	Within 24 hours of becoming aware of the event	SAE Report Form
Follow-Up Information on a Previously-Reported SAE	Within 72 hours of receiving the updated information	Updated SAE Report Form

SAE = serious adverse event.

11.5.2. Completion and Transmission of SAE Reports

Once an investigator becomes aware that a patient has experienced an SAE, he/she will report the information to Gemin X or its designee within 24 hours as outlined in Section 11.5.1. The SAE Report Form should always be completed as thoroughly as possible at the time of the initial report. If the investigator cannot complete the form in its entirety (i.e., not all of the information regarding the SAE is available), the report should be completed with as much information as possible and forwarded to Gemin X or its designee. All initial reports should contain, at a minimum, the following information:

- Investigator Name and Site Number
- Patient Identification
- The event or an initial description of the event
- Causality

If the causality is not known at the time of report transmission, the investigator is to offer his/her best medical judgment with the information available at that time, as described in Section 11.2.

The preferred method of submission of SAE reports to Gemin X or its designee is via facsimile to the attention of the Gemin X project contact. In rare circumstances and in the absence of facsimile equipment, notification by telephone is acceptable, with a copy of the SAE Report Form sent by overnight courier service. Initial notification via the telephone does not negate the need for the investigator to complete and sign the SAE Report Form within the timeframes specified in Section 11.5.1.

Please refer to the Contact Information page for a list of project contacts, telephone and fax numbers, as well as mailing addresses for SAE submissions.

11.5.3. Regulatory Reporting Requirements for SAEs

The investigator will promptly report all SAEs to Gemin X or its designee in accordance with the procedures detailed in Section 11.5.1. Gemin X has a legal responsibility to notify, as appropriate, both the local regulatory authority and other regulatory agencies about the safety of a product under clinical investigation. Prompt notification of SAEs by the investigator to the appropriate project contact for SAE receipt is essential so that legal obligations and ethical responsibilities towards the safety of other patients are met.

The investigator, or responsible person according to local requirements, will comply with applicable local regulatory requirements related to the reporting of SAEs to regulatory authorities and the Institutional Review Board (IRB).

This protocol has been filed under an Investigational New Drug (IND) application with the United States Food and Drug Administration (FDA). This protocol may also be conducted in regions outside of the United States and subject to clinical trial authorization by appropriate regulatory authorities. A given SAE may qualify as a Safety Report if the SAE is both attributable to the investigational product and unexpected. In this case, all investigators filed to the IND and other clinical trial applications will receive a copy of the Safety Report.

Safety Reports are prepared according to Gemin X policy and are forwarded to investigators as necessary. A Safety Report is prepared for an SAE that is both attributable to the investigational product and unexpected. The purpose of the Safety Report is to fulfill specific regulatory and Good Clinical Practice (GCP) requirements regarding the product under investigation.

When a patient experiences an SAE, the investigational staff is required to complete the necessary information on the SAE Report Form and fax it to Gemin X or its designee according to the submission requirements detailed in Section 11.5.1. Any subsequent queries received by the investigational staff from Gemin X or its designee should be answered and returned with any requested source documentation as soon as possible. Responses to queries should be made directly on the original form and faxed to Gemin X or its designee. All clarifications and/or corrections made by the investigational staff must be clearly identifiable and include initialing and dating of any changes.

NOTE: When submitting requested source documentation, investigational sites are required to remove any information that may identify the patient according to the Health Insurance Portability and Accountability Act (HIPAA) and other applicable standards of privacy protection. Sites are asked to obliterate any identifying information and to mark the copies of the source documents that are forwarded to Gemin X or its designee with the study patient identification information (i.e., patient initials and patient number).

From these SAE Report Forms, Safety Reports are generated and distributed to investigational sites. When a site receives from Gemin X an initial or follow-up Safety Report or other safety information (e.g., revised IB), the responsible person is required to promptly notify his/her IRB according to local requirements.

11.6. Follow Up of Adverse Events

After the initial report of an AE/SAE, the investigator is required to proactively follow each patient and provide further information to Gemin X or its designee on the patient’s condition.

All AEs and SAEs documented at previous visits/contacts and designated as ongoing will be reviewed at subsequent visits/contacts.

All AEs and SAEs will be followed until resolution, until the condition stabilizes, until the event is otherwise explained, or until the patient is lost to follow up. Once resolved, the appropriate

CRF pages and/or SAE Report Forms will be updated accordingly. The investigator will ensure that follow up includes any supplemental investigations as may be indicated to elucidate the nature and/or causality of the AE or SAE. This may include additional laboratory tests or investigations, histopathological examinations, or consultation with other health-care professionals.

Gemin X or its designee may request that the investigator perform or arrange for the conduct of supplemental measurements and/or evaluations to elucidate as fully as possible the nature and/or causality of the AE or SAE. The investigator is obligated to assist. If a patient dies during participation in the study or during a recognized follow-up period, Gemin X or its designee will be provided with a copy of any post-mortem findings, including histopathology.

New or updated information for a previously-reported event are to be submitted as a follow-up report on a new (blank) SAE Report Form, with only the changed information addressed and completed. However, the date the investigator became aware of the event and the patient identifier must be the same as provided on the initial report. The updated SAE Report Form should be resent to Gemin X or its designee within the timeframes outlined in Section 11.5.1.

11.6.1. Post-Study AEs and SAEs

A post-study AE/SAE is defined as any event that occurs outside of the AE/SAE detection period as defined in Section 11.6.1.

Investigators are not obligated to actively seek AEs or SAEs in former study participants. However, any AE/SAE that the investigator learns of, including those that lead to death, that occurs more than 28 days after the final dose of study drug and for which the investigator assesses as possibly, probably, or definitely related to the investigational product should also be reported.

11.6.2. SAEs Related to Study Participation

An SAE considered related to study participation (e.g., procedures, invasive tests, a change in existing therapy), even if it occurs during the pre- or post-treatment period, must be reported promptly to Gemin X or its designee (See Section 11.5.1).

12. PHARMACOKINETIC ASSESSMENT

12.1. Blood Samples

During Cycle 1 only, blood samples will be obtained from every patient for the determination of plasma concentrations of GX15-070. At each time point, 3 mL blood will be drawn into pre-chilled EDTA/K2 plastic tubes.

All samples will be kept on an ice-water slurry (~4°C) immediately after sampling. Within 15 min of collection, samples will be centrifuged at 4°C to yield plasma (1-2 mL). The plasma samples for PK analysis will be immediately frozen on dry ice, in a borosilicate glass container, and stored at –80°C prior to shipping to the Sponsor-designated laboratory for analysis.

For the 3-hour infusions, the total volume of blood sampled will be up to 63 mL over 3 days of dosing during Cycle 1. For the 24-hour infusions, the total volume of blood sampled will be up to 42 mL over 3 days of dosing during Cycle 1.

12.2. Sampling Times

For all patients receiving 3-hour obatoclax infusions, 21 blood samples will be drawn at the following points, during Cycle 1 only:

Day 1 timed from the start of the infusion: predose, 0.5, 1, 2.5, 3 (prior to end of infusion),

Day 1 timed from the end of the infusion: 0.5, 1, 3, and 9 hours,

Day 2: predose and 2.5 hours (30 minutes prior to end of infusion),

Day 3 timed from the start of the infusion: predose, 0.5, 1, 2.5, 3 (prior to end of infusion),

Day 3 timed from the end of the infusion: 0.5, 1, 3, 9, and 21 hours.

For all patients receiving 24-hour obatoclax infusions, 14 blood samples will be collected during Cycle 1 only. Blood samples will be drawn at: pre-dose, 1, 4, 8, 22, 32, 46, 56, and 70 hours (timed from the start of the infusion). Following termination of the 72-hour infusion period of Cycle 1, additional samples will be drawn at 0.5, 1, 3, 9, and 21 hours, timed from the end of the infusion.

Additional samples may be obtained at the discretion of the investigator if toxicities are observed. Not all PK samples will be collected from every patient if issues related to scheduling, timing, or other reasons interfere with the sampling process.

12.3. Technical Procedure

The concentration of GX15-070 in plasma will be determined using a validated assay.

12.4. Bioanalytical Method

The following parameters will be determined for each patient:

- Concentration of GX15-070 in each sample
- Concentration at end of infusion, area under the plasma concentration versus time curve (AUC), $AUC_{0\text{-}24h}$, AUC_{last}, half-life, clearance (Cl), and volume of distribution at steady state (V_{ss})

Mean ± standard deviation (percent coefficient of variability) will be determined.

13. STATISTICS

The primary objective of this study is to:

- Determine the rate of morphologic CR of obatoclax single-agent therapy in older patients with previously untreated AML.

The secondary objectives of this study are to:

- Based on the safety and efficacy profile of obatoclax in the pilot schedule evaluation, determine whether 3-hour or 24-hour obatoclax infusions should be utilized in the Phase II portion of the trial.
- Describe the duration of morphologic CR following treatment with obatoclax.
- Describe the safety profile of obatoclax administered every 2 weeks to older patients with previously untreated AML.
- Describe the mortality rate within 30 days of the first dose of obatoclax.
- Describe the utilization of red blood cell and platelet transfusions following the start of obatoclax.
- Characterize the PK profile of obatoclax administered every 2 weeks to older patients with previously untreated AML.
- Characterize baseline expression of bcl-2 family members in leukemic cells to investigate the hypothesis that increased expression of specific pro-survival family members or decreased expression of bax and bak correlates with response to obatoclax.

The Phase II portion of the trial will follow a 2-stage design powered to detect a true CR rate of ≥15% against a non-interesting rate of 5%, with an α=0.05 and a power of 90% [Simon 1989]. The formal 2-stage design will enroll 37 patients into Stage 1 using the obatoclax administration schedule determined to be favored in the pilot schedule evaluation. If, at the end of Stage 1 there are ≥3 CRs noted among the 37 patients enrolled, the trial will proceed to Stage 2, which will enroll an additional 47 patients for a total of up to 84. A total of 8 or more CRs out of the 84 patients enrolled into Stages 1 and 2 would be required to conclude that the CR rate is significantly greater than 5% with over 90% confidence and that the confidence intervals for the true CR rate include 15%.

The demographic, exposure, safety (AEs and clinical laboratory evaluations), efficacy, and PK data will be summarized across subsets. Tabulations will be produced for appropriate parameters. For categorical variables, summary tabulations of the number and percentage within each category (with a category for missing data) of the parameter will be presented. For continuous variables, the mean, median, standard deviation, minimum, and maximum values will be presented.

Response to treatment primarily will be assessed by the investigator based on peripheral blood counts that will be monitored throughout the study and bone marrow aspirates and biopsies that

will be repeated as necessary. The primary analysis of efficacy will be conducted on the rate of morphologic CR, as determined by an independent central pathologist. Additional, secondary efficacy analyses will be performed on the duration of complete response, the rate of partial remission, and the utilization of red blood cell and platelet transfusions following the start of obatoclax. Remission-free survival and overall survival will also be analyzed.

The rate of CR will be tabulated and accompanied by a 2-sided, 95% confidence interval. The duration of CR, defined as the duration in days from the date of first assessment of CR to the date of relapse or death, will be analyzed using Kaplan-Meier methods, with statistical assessment based on the log-rank statistic at the $\alpha=0.05$ significance level. The rates of CR will be calculated for first stage of the Phase II portion of the study as well as for the entire Phase II portion of the study.

Safety evaluation will be based on the incidence, intensity, and type of AEs, as well as clinically significant changes in physical examination findings, vital signs, and clinical laboratory results. Safety variables will be tabulated and presented for all subjects who receive any amount of study treatment. Exposure to study drug and reasons for discontinuation of study treatment will be tabulated. AEs will be coded using the Medical Dictionary of Regulatory Activities (MedDRA) AE coding system for purposes of summarization. All AEs occurring on study will be listed in by-subject data listings. Treatment-emergent events will be tabulated, where treatment-emergent is defined as any AE that occurs after administration of the first dose of study treatment through the end of treatment visit, any event that is considered drug-related regardless of the start date of the event, or any event that is present at baseline but worsens in intensity or is subsequently considered drug-related by the investigator. Events that are considered related to treatment (possibly, probably or definitely drug-related) will also be tabulated. AEs will also be tabulated by maximum severity. All SAEs including deaths will be documented through the end of treatment visit or 28 days after administration of the last dose of study treatment, whichever is later. Deaths, SAEs, and events resulting in study discontinuation will be tabulated.

Change from baseline in clinical laboratory parameters will be summarized across time on study. Shift tables will be produced for selected laboratory parameters, to include at least hemoglobin, white blood cell count, absolute neutrophil count, lymphocytes, platelets, ALT, AST, total bilirubin, creatinine, and electrolytes. These tables will summarize the number of subjects with each baseline NCI CTCAE grade and changes to the worst NCI CTCAE grade during study.

Changes in vital sign parameters will be summarized over time in a similar fashion to laboratory parameters, and any abnormal values will be tabulated.

PK parameters (maximum concentration [C_{max}], AUC, Cl, etc.) will be summarized by displaying means, standard deviations, and ranges. Exploratory relationships between PK parameters and population characteristics will be performed as appropriate.

14. DIRECT ACCESS TO SOURCE DATA/DOCUMENTS

14.1. Study Monitoring

In accordance with applicable regulations, GCP guidelines, and Gemin X policy, sites will be contacted by Gemin X or its designee prior to patient enrollment to ensure the adequacy of the investigational site, review the protocol and data-collection procedures, review the investigational staff's responsibilities, and review the study-administrative procedures with the investigator and staff.

In addition, Gemin X or its designee will periodically perform on-site inspections (routine monitoring visits), the frequency of which will be based on such considerations as the study objective and/or endpoints, the purpose of the study, study-design complexity, and enrollment rate.

During these visits, Gemin X or its designee will:

- Check the progress of the study.
- Conduct source document verification.
- Review the study data and process CRFs (electronic or paper).
- Inspect regulatory documentation including patient informed consent forms.
- Inspect the pharmacy and study-drug inventory.
- Identify any issues and address their resolution.

This will be done in order to verify that the:

- Data are authentic, accurate, and complete.
- Safety and rights of patients are being protected.
- Study is being conducted in accordance with the currently approved protocol (which also includes any amendments), GCP, and all other applicable regulatory requirements.

Gemin X or its designee will provide CRFs (electronic for paper) for each patient. If data will be collected using electronic CRFs, Gemin X or its designee will not provide paper CRFs. However, Gemin X or its designee will provide source document worksheets if requested by the site. Each patient will be identified by a study identification number and patient initials. A patient should never be identified by name or any other identifier not approved by HIPAA guidelines for patient privacy.

If using paper CRFs, black ink should be used for all data entries into the CRF and when completing regulatory documentation. Errors should be corrected by striking the information with 1 line, writing the correct information next to the appropriate data field, and initialing and

dating the change. Incorrect information entered should not be obliterated when corrected. When a patient completes his/her participation in the study, CRFs should be completed in a timely fashion and signed on the appropriate signature pages by the investigator. Electronic errors will be captured by the audit trail within the electronic system.

CRFs, (electronic for paper) all copies of test results, and all study-related regulatory documents must be available at all times for Gemin X or its designee or regulatory agency inspection. During the periodic site monitoring visits, the source documents will be verified against data entered onto the CRF (electronic for paper) in order to assure that all data is accurately and completely reflected. The investigator must agree to allocate his/her time and the time of his/her staff to Gemin X or its designee during the monitoring visits in order to discuss findings and any relevant issues.

At study closure, Gemin X or its designee will also conduct all activities described in Section 17.4.

14.2. Audits and Inspections

See Section 15.

14.3. Institutional Review Board

The protocol, informed consent form, and any other information that will be presented to potential patients for this study (e.g., advertisements or information that supports or supplements the informed consent) must be reviewed and approved by an appropriately accredited IRB as defined by GCP/ International Conference on Harmonization (ICH) guidelines. The list of the IRB-voting members, their duties, titles or occupations, and their institutional and IRB affiliations must be submitted to Gemin X or its designee for review and approval prior to shipment of drug supplies to the investigator. A multiple assurance number with appropriate documentation from the Office of Health and Human Services is also acceptable in lieu of an IRB membership list.

A copy of the approval letter from the IRB must be received by Gemin X or its designee prior to shipment of drug supplies to the investigator. The approval letter must contain, at a minimum, the protocol title and/or number, the investigator's name, and the date of approval.

Changes to the protocol and informed consent form must also be submitted to the IRB for review and approval. Any new or revised IRB-approval letters must be forwarded to Gemin X or its designee promptly. The investigator must follow all applicable regulatory requirements pertaining to the use of an amended informed consent form before obtaining any new patients' consent to participate in the study. Records of IRB annual review, and if applicable, approval of all documents pertaining to this study must be sent to Gemin X or its designee and kept on file with the investigator. These documents are subject to inspection by regulatory authorities, Gemin X or its designee at any time during the study and for 2 years following approval of the

last marketing application for the drug. If the drug is not approved, these records must be retained for 2 years following discontinuation of clinical drug shipments.

14.4. Investigator Reporting Requirements

As indicated in Section 11.5.3, the investigator (or Sponsor, where applicable) is responsible for reporting all SAEs to the IRB in accordance with all applicable regulations. Periodic status reports and AE updates that are required by the IRB are the responsibility of the investigator and must be submitted to the IRB according to the IRB's reporting requirements. The IRB must also be notified of the completion of the study, and a final report must be submitted in accordance with the IRB's reporting policies. A copy of these reports should also be forwarded to Gemin X or its designee upon completion. The investigator must maintain an accurate record of all communication, reports, and submissions to the IRB.

15. QUALITY CONTROL AND QUALITY ASSURANCE

To ensure compliance with GCP and all applicable regulatory requirements, Gemin X or its designee may conduct a quality assurance audit. Regulatory agencies may also conduct a regulatory inspection of this study. Such audits/inspections can occur at any time during or after completion of the study. If an audit or inspection occurs, the investigator and investigational site agree to allow the auditor/inspector direct access to all relevant documents and to allocate his/her time and the time of his/her staff to the auditor/inspector to discuss findings and any relevant issues.

16. ETHICS

16.1. Ethics Review

Gemin X or its designee will obtain approval to conduct the study from the United States FDA, Health Canada, and/or other responsible regulatory agency in accordance with any applicable regulatory requirements prior to initiating the study at any investigational site in North America or Europe.

16.2. Ethical Conduct of the Study

This study will be conducted in accordance with local, state, and federal regulations, as well as GCP and ICH guidelines and the October 2000 version of the Declaration of Helsinki.

The investigator will be responsible for the overall conduct of the clinical trial for the site and is to ensure that the study is conducted according to the protocol and all applicable regulatory requirements and IRB regulations. The following must be observed to comply with local, state, and federal regulations as well as Gemin X guidelines and standards.

16.3. Written Informed Consent

Informed consent must be obtained by the investigator (or staff designee) before any patients can participate in the study. After the investigator (or staff designee) has fully explained the trial to the patient, the patient has had time to consider the information fully, and has been encouraged to ask questions, the patient will be asked to give consent to participate in the clinical trial by signing the informed consent form. This consent process must be witnessed, signed, dated, and retained by the investigator as part of the study records. A copy of the fully-executed informed consent form must be given to the patient immediately. If an Experimental Subject's Bill of Rights is applicable in the investigator's state, that form must also be prepared and signed by each patient and retained by the investigator as part of the study records. A copy of the Bill of Rights must also be given to the patient.

The contents of the informed consent form must be in accordance with all applicable local, state, and federal regulations and contain the following basic elements:

1. A statement that the study involves research, an explanation of the purposes of the research and the expected duration of the patient's participation, a description of the procedures to be followed, and identification of any procedures that are experimental.
2. A description of any reasonably foreseeable risks or discomforts to the patient.
3. A description of any benefits to the patient or to others which may reasonably be expected from the research.
4. A disclosure of appropriate alternative procedures or courses of treatment, if any, that might be advantageous to the patient.

5. A statement describing the extent, if any, to which confidentiality of the records identifying the patient will be maintained and that notes the possibility that the regulatory authority and Sponsor (Gemin X or its designee) may inspect the records.
6. For research involving more than minimal risk, an explanation as to whether any compensation or medical treatments are available if injury occurs and, if so, what they consist of or where further information can be obtained.
7. An explanation of whom to contact for pertinent questions about the research and research patient's rights, and whom to contact in the event of a research-related injury to the patient.
8. A statement that participation is voluntary, that refusal to participate will involve no penalty or loss of benefits to which the patient is otherwise entitled, and that the patient may discontinue participation at any time without penalty or loss of benefits to which the patient is otherwise entitled.
9. A statement that the particular treatment or procedure may involve risks to the patient (or to the embryo or fetus, if the patient is or may become pregnant), which are currently unforeseeable.
10. Anticipated circumstances (including termination by the Sponsor) under which the patient's participation may be terminated by the investigator without regard to the patient's consent.
11. Any additional costs to the patient that may result from participation in the research.
12. The consequences of a patient's decision to withdraw from the research and procedures for orderly termination of participation by the patient.
13. A statement that significant new findings developed during the course of the research that may relate to the patient's willingness to continue participation will be provided to the patient.
14. The approximate number of patients involved in the study.

Additionally, informed consent forms will not include any exculpatory language through which the patient is made to waive or appear to waive any legal rights. In obtaining informed consent, all information presented to patients will be in layman's terms or in a language that is understandable to the patient.

17. DATA HANDLING AND RECORDKEEPING

Gemin X retains exclusive ownership of all data, results, reports, findings, discoveries, and any other information collected during this study. Investigators and investigational institutions are to refer to their respective individually negotiated Clinical Trial Agreement for rights and restrictions regarding publication, ownership, inventions, disclosure, and confidentiality.

17.1. Inspection of Records

The Sponsor's clinical monitor or designee will maintain contact with the investigator by telephone, letter, and visits to evaluate study and protocol conduct. The investigator will allow the Sponsor's monitors to inspect the clinical, laboratory, and pharmacy facilities to assure compliance with GCP and Good Laboratory Practices.

The CRFs and corresponding original patient medical records (source documents) are to be fully available for review by the Sponsor's representatives at regular intervals. These reviews verify adherence to the study protocol and data accuracy in accordance with federal regulations. The study site and study records are subject to inspection by the FDA.

17.2. Retention of Records

All documents relating to the clinical trial, including the protocol and IB, are the confidential property of Gemin X and should be regarded as such. Unused clinical-trial materials such as CRFs and/or source document worksheets should be returned to Gemin X or its designee at the time of study closure.

Following closure of the study, the investigator must retain all site study records in a safe and secure location that is easily accessible for inspection for a period of 2 years following the last approval of a marketing application of the compound in an ICH region. If the drug does not receive marketing approval, these records must be retained for a period of 2 years following cessation of clinical drug shipment. If the investigator retires or changes employment, custody of the records may be transferred to another suitable person who will accept responsibility for those records. If this change occurs, Gemin X or its designee must be informed in writing by the investigator prior to his/her departure.

17.3. Data Management

The main objective is to obtain those data required by the study protocol in a complete, accurate, legible, and timely fashion. The data in either a paper or electronic CRF should be consistent with the relevant source documents.

The data recorded in the course of this study must be documented in the CRFs and/or SAE Report Form, and must be forwarded to the sponsor/designee. Data entered into the electronic data capture system will be immediately saved to a central database and changes tracked to

provide an audit trail. They shall then be processed, evaluated, and stored in anonymous form in accordance with the data protection regulations.

The investigator must ensure that the CRFs forwarded to the sponsor/designee and any other associated documents contain no mention of any subject names.

All data in this study are expected to be collected in an electronic CRF using an electronic data capture system, which is compatible with 21 CFR, part 11 requirements. In the event paper CRFs are used, they must be filled in completely and legibly (with a black ballpoint pen, acceptable for use on official documents). Any amendments and corrections necessary must be undertaken and countersigned by the investigator or designee, stating the date of the amendment/correction and initials. When complete, the original CRFs will be collected, and a carbon copy will be left with the investigator as part of the site study files. At the end of the study, the investigator will sign-off on their completed CRFs documenting that to the best of his/her knowledge, all recorded data is accurate and can be verified in the corresponding medical source documents. Any written errors must remain legible and may not be deleted with correction aids. Electronic errors will be captured by the audit trail within the electronic system. The subject's medical source documents must support any changes made.

In the case of missing data/remarks, the entry spaces provided for in the CRF should be cancelled out so as to avoid unnecessary follow-up inquiries. Further details on CRF completion are handled in the CRF completion instructions.

The CRFs (electronic or paper) are official documents and must be suitable for submission to authorities. The investigator must keep a written or electronic subject file for every subject participating in the clinical study. It must be possible to identify each subject by the subject's study number using the subject file.

Documents related to the clinical trial including original subject medical records, must be retained by the investigator/institution to satisfy regulatory requirements. When data have been entered, reviewed, edited, and source data verification performed, the principal investigator will be notified to sign the electronic CRF electronically as per the agreed project process and the data will be locked to prevent further editing. A copy of the electronic CRF will be archived at the study site.

17.4. Study and Site Closure

Upon completion of the study, Gemin X or its designee will conduct the following activities in conjunction with the investigator or site staff, as appropriate:

- Return of all study data to Gemin X or its designee.
- Review and complete data queries with the study staff.
- Perform study-drug accountability, inventory-record reconciliation, and make arrangements for the disposal of unused investigational product(s).
- Review of site study records for completeness.

In addition, Gemin X reserves the right to temporarily suspend or prematurely discontinue this study either at a single site or at all sites at any time for reasons including, but not limited to, safety or ethical issues or severe non-compliance. If Gemin X determines such action is needed, Gemin X will discuss this with the investigator (including the reasons for taking such action) at that time. When feasible, Gemin X will provide advance notification to the investigator of the impending action prior to it taking effect.

Gemin X or its designee will promptly inform all other investigators and/or investigational sites conducting the study if the study is suspended or terminated for safety reasons, and will also inform the regulatory authorities of the suspension or termination of the study and the reason(s) for the action. If required by applicable regulations, the investigator must inform the IRB promptly and provide the reason for the suspension or termination.

If the study is prematurely discontinued, all study data must be returned to Gemin X. In addition, arrangements will be made for all unused investigational product(s) in accordance with the applicable Gemin X procedures for the study.

Financial compensation to investigators and/or investigational sites will be in accordance with the Clinical Trial Agreement established between the investigator and Gemin X.

17.5. Provision of Study Results and Information to Investigators

Gemin X is committed to full and rapid reporting of its clinical research. Following the completion of the clinical trial, a clinical study report will be generated and any major findings of the study will be provided to the principal investigator.

18. REFERENCES

Anderson JE, Kopecky KJ, Wiillman CL, et al. Outcome after induction chemotherapy for older patients with acute myeloid leukemia is not improved with mitoxantrone and etoposide compared to cytarabine and daunorubicin: a Southwest Oncology Group study. *Blood.* 2002:100;2869-3876.

Appelbaum FR, Gundacker H, Head DR, et al. Age and acute myeloid leukemia. *Blood.* 2006;107:3481-3485.

Beauparlant P, Shore GC. Therapeutic activation of caspases in cancer: A question of slectivity. *Current Opinion in Drug Discovery & Development.* 2003;6:179-87.

Cheson BD, Bennett JM, Kopecky KJ, et al. Revised recommendations of the International Working Group for Diagnosis, Standardization of response criteria, treatment outcomes, and reporting standards for therapeutic trials in acute myeloid leukemia. *J Clin Oncology*. 2003;21(24):4642-4649.

Cory S, Adams JM. The BCL2 Family: regulators of the cellular life-or-death switch. *Nature Reviews Cancer*. 2002;2:647-56.

Frohling S, Schlenk RF, Kayser S, et al. Cytogenetics and age are major determinants of outcome in intensively treated acute myeloid leukemia patients older than 60 years: results from AMLSG trial AML HD98-B. *Blood*. 2006;108;3280-3288.

Godwin JE, Kopecky KJ, Head DR, et al. A double-blind placebo-controlled trial of granulocyte colony-stimulating factor in elderly patients with previously untreated acute myeloid leukemia: A Southwest Oncology Group Study. *Blood.* 1998;91:3607-3615.

Menzin J, Lang K, Earle CC, et al. *Arch Intern Med*. 2002;162:1597-1603.

Reed JC. Apoptosis-targeted therapies for cancer. *Cancer Cell*. 2003;3:17-22.

Simon, R. Optimal Two-Stage Designs for Phase II Clinical Trials. *Controlled Clinical Trials*.1989;10:1-10.

Stone RM. Older AML patients: fit for what? Blood. 2006;108:3232-3222.

Tallman MS, Gilliland DG, Rowe JM. Drug therapy for acute myeloid leukemia. *Blood*. 2005;106:1154-1163.

Taylor PRA, Reid MM, Stzrk AN, et al. De novo acute myeloid leukemia in patients over 55 years old: A population-based study of incidence, treatment, and outcome. *Leukemia*. 1995;9:231-237.

Watt JC, Konopleva M, Contractor R, et al. Inhibition of Bcl-2 signaling by small molecule BH3 inhibitor GX15-070 as a novel therapeutic strategy in AML. *Blood.* 2006;108:728a.

19. APPENDICES

19.1. Appendix 1: Study Design Logic Diagram

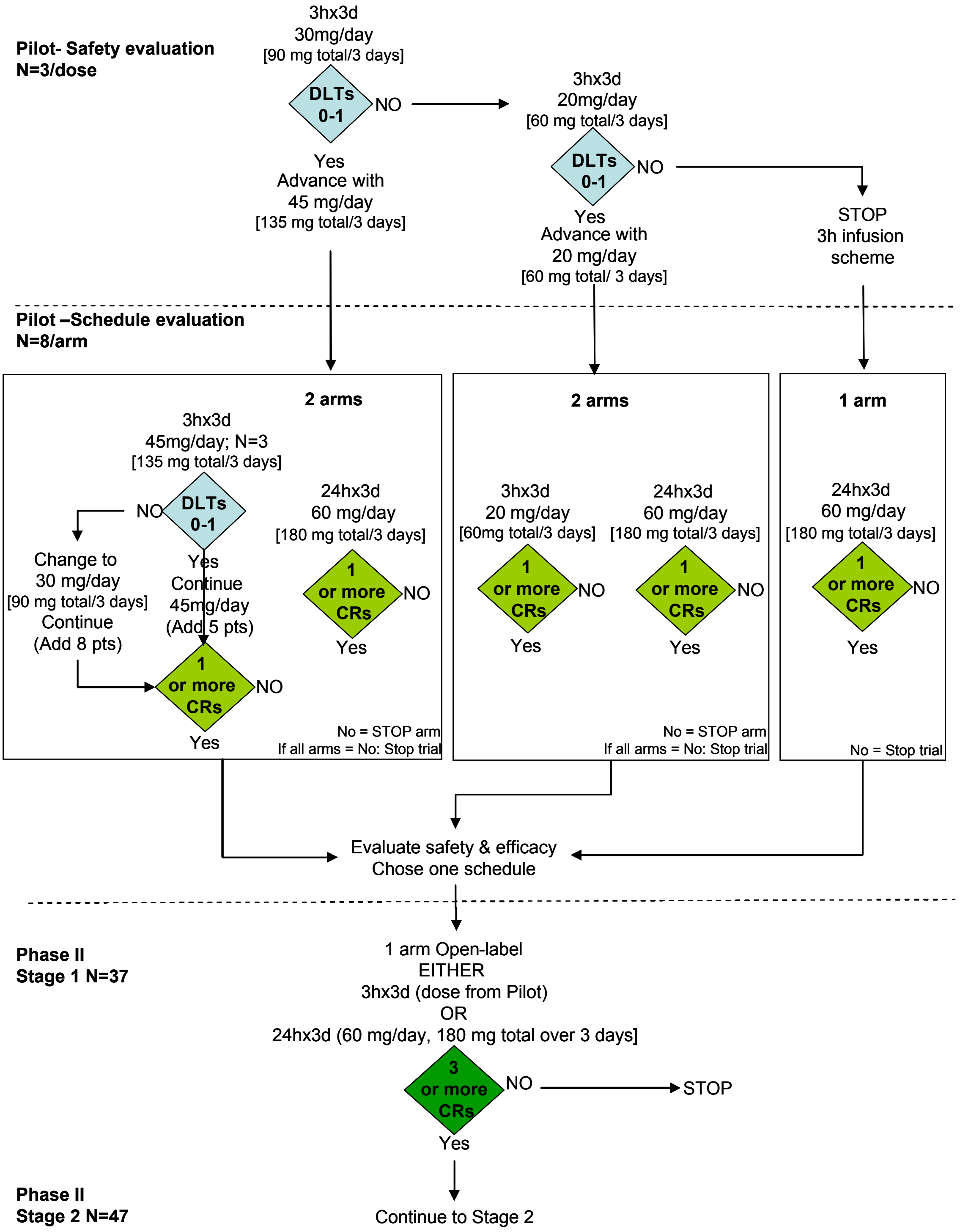

19.2. Appendix 2: ECOG Performance Scale

Grade	ECOG Performance Status
0	Fully active, able to carry on all predisease performance without restriction.
1	Restricted in physically strenuous activity, but ambulatory and able to carry out work of a light or sedentary nature, e.g., light house work, office work.
2	Ambulatory and capable of all selfcare, but unable to carry out any work activities. Up and about more than 50% of waking hours.
3	Capable of only limited selfcare, confined to bed or chair more than 50% of waking hours.
4	Completely disabled. Cannot carry on any selfcare. Totally confined to bed or chair.
5	Dead

Signature Page

This document was approved by:		
**Status**	**Date**	**Name - Title - Department**
Approved	21/12/2007 13:40:02	Berger Mark - VP of Clinical Development - Clinical Development
Comment		
Approved	21/12/2007 15:10:21	Mariani Mark - Senior Director - Regulatory Affairs and Compliance
Comment		

This document was authorized by:		
**Status**	**Date**	**Name - Title - Department**
Authorized	21/12/2007 15:12:44	Berger Mark - VP of Clinical Development - Clinical Development
Comment		
